# Systematic review and meta-analysis of clinical effectiveness of self-management interventions in Parkinson’s disease

**DOI:** 10.1186/s12877-021-02656-2

**Published:** 2022-01-11

**Authors:** Jennifer S. Pigott, Edward J. Kane, Gareth Ambler, Kate Walters, Anette Schrag

**Affiliations:** 1 Queen Square Institute of Neurology, University College London, London, UK; 2 South London & Maudsley NHS Foundation Trust, London, UK; 3 Department of Statistical Science, University College London, London, UK; 4 Centre for Ageing Population Studies, Research Department of Primary Care and Population Health, University College London, London, UK; 5 UCL Institute of Neurology, Royal Free Campus, University College London, London, NW3 2PF UK

**Keywords:** Parkinson’s disease, Long-term health conditions, Neurodegenerative disease, Self-management, Self-care, Quality of life, Wellbeing, Activities of daily living, Functioning, Systematic review

## Abstract

**Background:**

Parkinson’s disease is a complex neurodegenerative condition with significant impact on quality of life (QoL), wellbeing and function. The objective of this review is to evaluate the clinical effectiveness of self-management interventions for people with Parkinson’s disease, taking a broad view of self-management and considering effects on QoL, wellbeing and function.

**Methods:**

Systematic searches of four databases (MEDLINE, Embase, PsycINFO, Web of Science) were conducted for studies evaluating self-management interventions for people with Parkinson’s disease published up to 16th November 2020. Original quantitative studies of adults with idiopathic Parkinson’s disease were included, whilst studies of atypical Parkinsonism were excluded. Full-text articles were independently assessed by two reviewers, with data extracted by one reviewer and reliability checked by a second reviewer, then synthesised through a narrative approach and, for sufficiently similar studies, a meta-analysis of effect size was conducted (using a random-effects meta-analysis with restricted maximum likelihood method pooled estimate). Interventions were subdivided into self-management components according to PRISMS Taxonomy. Risk of bias was examined with the Cochrane Risk of Bias 2 (RoB2) tool or ROBIN-I tool as appropriate.

**Results:**

Thirty-six studies were included, evaluating a diverse array of interventions and encompassing a range of study designs (RCT *n* = 19; non-randomised CT n = five; within subject pre- and post-intervention comparisons *n* = 12). A total of 2884 participants were assessed in studies across ten countries, with greatest output from North America (14 studies) and UK (six studies). Risk of bias was moderate to high for the majority of studies, mostly due to lack of participant blinding, which is not often practical for interventions of this nature. Only four studies reported statistically significant improvements in QoL, wellbeing or functional outcomes for the intervention compared to controls. These interventions were group-based self-management education and training programmes, either alone, combined with multi-disciplinary rehabilitation, or combined with Cognitive Behaviour Therapy; and a self-guided community-based exercise programme. Four of the RCTs evaluated sufficiently similar interventions and outcomes for meta-analysis: these were studies of self-management education and training programmes evaluating QoL (*n* = 478). Meta-analysis demonstrated no significant difference between the self-management and the control groups with a standardised mean difference (Hedges g) of − 0.17 (− 0.56, 0.21) *p* = 0.38. By the GRADE approach, the quality of this evidence was deemed “very low” and the effect of the intervention is therefore uncertain.

Components more frequently observed in effective interventions, as per PRISMS taxonomy analysis, were: information about resources; training or rehearsing psychological strategies; social support; and lifestyle advice and support. The applicability of these findings is weakened by the ambiguous and at times overlapping nature of self-management components.

**Conclusion:**

Approaches and outcomes to self-management interventions in Parkinson’s disease are heterogenous. There are insufficient high quality RCTs in this field to show effectiveness of self-management interventions in Parkinson’s disease. Whilst it is not possible to draw conclusions on specific intervention components that convey effectiveness, there are promising findings from some studies, which could be targeted in future evaluations.

**Supplementary Information:**

The online version contains supplementary material available at 10.1186/s12877-021-02656-2.

## GRADE summary of findings

**Table:** 

**Self-Management for people with Parkinson’s** **disease** **and their Caregivers**				
**Population:** people with idiopathic Parkinson’s disease and/or their caregivers **Intervention:** self-management **Comparison:** control arm (usual care or information only)				
**Outcomes**	**Illustrative Comparative Risks**		**No. of participants (studies)**	**Quality of the evidence (GRADE)**
	**Usual Care**	**Self-management**		
**Quality of Life** PDQ39 or custom questionnaire (follow-up range 3 weeks – 6 months)	See Comment*	See Comment*	478 (4)	⨁◯◯◯ VERY LOW Due to serious risk of bias, inconsistency, and imprecision.

For GRADE evidence profile, see Additional file 5

Note a further 32 studies are also discussed in this review, evaluating a range of clinical outcomes

* Mean values are not presented since 3 trials reported values for PDQ-39 whereas the 4th reported values from a custom questionnaire. Furthermore, 3 trials reported post-randomisation values, and the 4th reported ‘change’ values

## Introduction

Parkinson’s disease is a complex progressive neurological condition for which there is currently no cure. Its prevalence is rising [1], and increases with age [2]. Parkinson’s disease is associated with a range of motor and non-motor features that affect quality of life [3], but clinical reviews to improve these features may not be frequent enough to address these. In the last few years, there has therefore been increasing interest in the use of self-management approaches for features of Parkinson’s disease. Research in other long term conditions (LTCs) has provided evidence that supporting self-management can improve health and quality of life outcomes, and may decrease health care utilisation [4].

Core self-management skills include: problem solving, decision making, resource utilisation, forming of a patient/health care provider partnership, and taking action [5]. Self-management support interventions aim to develop these skills for people with LTCs. Key components of self-management support [6] have been defined through the PRISMS taxonomy, which comprises 14 components, e.g. “monitoring of condition with feedback” and “social support”. As these differ between LTC, condition-specific self-management interventions have emerged, with evidence that effective interventions are multifaceted and tailored to the individual [7].

In Parkinson’s disease, disease progression simultaneously increases illness demands and challenges an individual’s physical and cognitive capacity to adapt to such demands. The heterogeneity of experience of Parkinson’s disease makes a uniform approach difficult. However, recommendations for management exist based on evidence for improved outcomes for people with Parkinson’s disease, including medication, non-pharmacological therapy and exercise recommendations [8]. Patient choices in day-to-day life influence these, and are therefore potential targets for self-management.

A previous review of self-management approaches in Parkinson’s disease (2016) identified 18 interventions, 16 specifically for Parkinson’s disease, although studies targeting a single outcome were excluded [9]. The interventions varied in structure, content, and targeted outcomes. Evidence to support self-management programmes for Parkinson’s disease was found to be limited: Only 7 full-text studies were included, and only 1 was a randomised controlled trial (RCT). Others were identified conference abstracts (5 presenting data, 3 only descriptions of interventions), reviews or protocol papers. 39% of the interventions included the three key self-management components of education, goal setting and problem solving. Effective active components of interventions could not be determined, but the authors speculated that potential factors impacting effectiveness may be: intervention factors, such as caregiver involvement and peer-interaction, participant factors such as stage and cognitive condition, and, in the future, use of technology.

As there has been a substantial increase in studies in this field since 2016, we conducted a new systematic review and meta-analysis, considering data also from more recent studies and taking a broader view of self-management interventions, through wider inclusion criteria (see 2. Methods). This adds breadth to the range of interventions considered, reflective of the breadth of issues in Parkinson’s disease. Whilst not excluding on the basis of outcome measure, we particularly focus on quality of life (QoL), wellbeing and functional outcome measures in this review as a patient-centred approach for patient-focussed interventions [10].

## Methods

### Source of Data & Search Strategy

This review was conducted according to the Preferred Reporting Items for Systematic Reviews and Meta-analyses (PRISMA) guidelines [11, 12]. Small deviations from the guidelines have been described, along with rationale, in the relevant sections. The review protocol was registered on PROSPERO: CRD42019117183.

One reviewer conducted online searches in the following databases: MEDLINE, Embase, PsycINFO, Web of Science. They were searched from inception, initially to 31st October 2018 (EK), with an updated search on 16th November 2020 (JP). Searches were not restricted by date of publication. Forwards and backwards citation tracking of key articles to identify other relevant studies was conducted.

The search strategy involved a combination of Parkinson’s disease terms; “Parkinson” OR Parkinson’s Disease, AND self-management terms; Self-Management OR Self care. For the full search strategy see Additional file 1.

### Inclusion & Exclusion Criteria

The inclusion criteria are detailed in Table 1 using the PICOS format.

**Table 1:** Inclusion and Exclusion Criteria

	Inclusion	Exclusions	Comments
**Population**	Adult participants with idiopathic Parkinson’s disease, with or without their carers	Atypical Parkinsonism; articles where Parkinson’s disease data was indistinguishable from other conditions.	Diagnosis of Parkinson’s disease is widely reported based on the UK Parkinson’s Disease Society Brain Bank Diagnostic Criteria, requiring diagnosis of a parkinsonian syndrome, exclusion of other causes, and supportive features [13]. Carers have been shown to have an important role in management and supporting self-management of Parkinson’s disease [14] so were included to represent the care partnership.
**Intervention**	Self-management interventions		Interventions that train or were based on the individual utilising skills to manage “the symptoms, treatment, physical and psychological consequences and lifestyle changes inherent in living with a chronic condition” [15]
**Comparator**	Any		
**Outcome**	Any		Due to heterogeneity of Parkinson’s disease and targets of the self-management interventions, a range of primary outcome measures were anticipated and so included. The outcomes considered to be the ‘patient important outcomes’ for these interventions are Quality of Life (QoL), wellbeing and function (Activities of Daily Living) so are considered in more detail in this review.
**Studies**	Original quantitative studies	Expert opinions, letter to the editor, case-reports, editorials, reviews, conference abstracts without full report, and qualitative studies.	Qualitative data has been synthesised elsewhere [14]. The search was not restricted by date or language, but articles were not included if the full text was not available in English language.

Whilst there is no agreed single definition of self-management, we used Barlow et al’s definition: an “individual’s ability to manage the symptoms, treatment, physical and psychological consequences and lifestyle changes inherent in living with a chronic condition” [15]. Interventions self-defining as ‘self-management’ were checked against this definition. Additionally, where the term ‘self-management’ was not used, if the premise of the intervention was the individual managing their Parkinson’s disease symptoms, treatments or consequences, or being taught to do so, then the intervention was included. As a result, in the context of Parkinson’s disease, studies targeting self-management of specific clinical aspects, such as posture, and drooling, and those targeting self-management of specific treatments, such as exercises, were considered fulfilling the inclusion criteria.

### Study selection

One reviewer (EK for initial search, JP for update) screened all titles and abstracts of the identified studies in accordance with the inclusion and exclusion criteria. For those deemed eligible from screening, full texts were obtained and reviewed independently by both reviewers (EK and JP). Any discrepancies were discussed and resolved through consultation with a third and fourth reviewer (AS and KW).

### Data extraction

Data extraction was performed by one reviewer (JP) with a second reviewer (EK) conducting an independent reliability check. No discrepancies were identified. Data was extracted into a standardised form, including: lead author, publication date, country; population; study design; intervention type; sample size, age and stage of Parkinson’s disease; results for primary outcomes, and for secondary outcome measures of quality of life (QoL), wellbeing and function (Activities of Daily Living) measures. Articles reporting on the same intervention with overlapping samples were each included for data extraction.

### Data synthesis

Meta-analysis was undertaken for the studies using similar aims, interventions and outcomes. For the remainder, a narrative synthesis approach is taken. For meta-analysis, we estimated the standardised mean difference (Hedges g) and standard error from each study, then used random-effects meta-analysis with REML (restricted maximum likelihood) to estimate the pooled estimate. The I^2^ statistic was used to quantify heterogeneity. Statistical analyses were conducted using Stata 16.1.

## Quality

The RCTs were examined for bias using the Cochrane Risk of Bias 2 (RoB2) tool [16]. This tool assesses several key areas of potential bias: randomisation methods; deviations from intended intervention; missing data; measurement of outcome; and selection of reported result. The non-randomized studies of interventions (NRSIs) were assessed using the ROBIN-I tool [17]. This tool assesses risk of bias due to confounding; selection bias; classification of the intervention; deviations from intended intervention; missing data; measurement of outcome; and selection of reported result. A ‘Low’ risk of bias on ROBIN-I for NRSI is considered to mean the study is comparable to a well-performed RCT with regard to the specific domain. ‘Moderate’ risk of bias is considered to mean the study is sound for a non-randomized study with regard to this domain but cannot be considered comparable to a well-performed RCT. For both, the risk of bias was assessed against the specific outcome. The GRADE approach was followed and uncertainty assessment performed for studies included in meta-analysis [18].

## Results

### Study selection

**Fig. 1:**
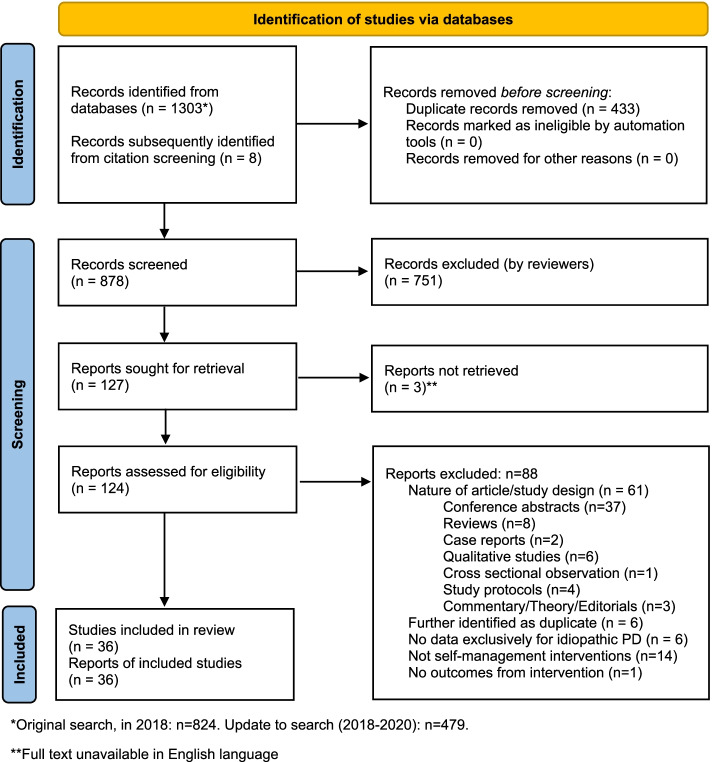
PRISMA Flow Diagram

As shown in Fig. 1, the online databases search yielded a total of 1303 articles: 824 in the original search and a further 479 articles through the update. Eight additional articles were identified through citation screening. Removal of duplicates left 878 articles. Through screening of abstracts, 127 articles were found to be eligible for full text review, three of which were unavailable in English.  By means of independent full review, both reviewers agreed on exclusion of 88 articles with reasons, leaving the inclusion of 36 articles in the final review. Reasons for exclusion were: nature of article or study design (*n* = 61); interventions were not self-management (*n* = 14); duplicates (*n* = 6); no Parkinson’s disease specific data presented (*n* = 6); description of intervention without outcome measures (*n* = 1).

### Quality assessment

The results of quality assessment are summarised in Table 2 (see Additional file 3 for full assessment results). Risk of Bias was moderate to high for almost all included studies. The main determinant for bias is the self-reported nature of the outcome measures combined with lack of participant blinding. This concern was almost universal as relates to the nature of these interventions – participants know whether or not they have received the intervention, and so lower risk of bias would not be possible to achieve. The only studies achieving low risk of bias used active controls to achieve participant blinding to allocation. Deviations from protocol were rare, but missing data due to participant drop-out was not uncommon.

**Table 2:** Study Characteristics & Results

**Study** Author, Year, Country	**Population** & notable exclusions	**Sample** Participants (n); Age; H&Y Stage	**Intervention & Comparator**	**Outcome** Primary (underlined) & Relevant Secondary (Measure)	**Results**		**Risk of Bias**
**Design: Randomised Controlled Trials**							
Dobkin [19], 2020 USA	People with PD + depression (current); age 35-85 yr; stable condition; family/friend willing to participate. Excluded: MoCA< 21, medically unstable or primary psychotic/bipolar/substance abuse disorder.	*n* = 72 Mean age 65 yrs. H&Y not given	PD-informed telephone-Cognitive Behaviour Therapy (T-CBT) with self-management in addition to ‘enhanced TAU’ vs ‘Enhanced’ treatment as usual: TAU+ clinical monitoring by study staff & provision of a resource list.	Difference in mean improvement CBT vs TAU (95% CI)			Some concerns
				Depression (HAM-D)	6.88 (4.73–9.03) at end of intervention 5.15 (2.99–7.31) at 6 month follow-up F_4,249_ = 14.89, ***p*** **< 0.0001** at end of intervention; and at 6-month follow-up (***p*** **< 0.0001**)		
				QoL (Mental Health composite Score (MCS) of the SF-36)	4.48 (−0.86 to 9.83) at end of intervention 4.70 (− 0.64 to 10.04) at end of 6 month follow-up F4,241 = 3.62, ***p*** **= 0.007**		
				*Other reported outcomes:* Responder status (Clinical Global Impression Improvement Scale); Depression severity (BDI); Anxiety (HAM-A); Negative thinking (Inference Questionnaire)			
Navarta-Sanchez, [20] 2020 Spain (cluster randomisation)	People with PD (any stage), fluent in Spanish, and their informal caregivers Excluded: Cognitive impairment.	*n* = 140 (PD) + 127 (carers) Mean age, PD: 75 yrs. (intervention) & 72 yrs. (control). Mean age, carers: 67 yrs. (intervention) & 64 yrs. (control). H&Y range I-V (majority I-III)	Psychoeducation vs Control: education only	Mean (SD) pre/post/6 months follow-up for intervention vs control			High
				QoL (PDQ-39)	Pre 21.38 (14.12), post 20.42 (14.78), 6 m 24.61 (18.54) vs pre 19.44 (12.17), post 17.05 (12.87), 6 m 23.69 (14.92) Time effect 8.49 (***p*** **< 0.001**), time*group interaction 0.59 (*p* = 0.554)		
				Caregiver QoL (SQLC)	Pre 119.11 (22.55), post 120.39 (23.68), 6 m 119.64 (21.86) vs pre 117.83 (23.49), post 117.02 (23.57), 6 m 114.00 (27.33) Time effect 0.96 (*p* = 0.386), Time*group interaction 1.89 (*p* = 0.157)		
				Psychosocial adjustment (PAIS-SR)	People with PD: Pre 35.05 (16.90), post 32.29 (16.42), 6 m 37.80 (18.34) vs pre 34.12 (19.59), post 30.68 (17.72), 6 m 37.82 (17.34) Time effect 8.28 (***p*** **= 0.001**), Time*group interaction 0.14 (*p* = 0.868) Caregivers: Pre 32.41 (16.33), post 27.70 (14.51), 6 m 30.70 (13.04) vs pre (28.31 (17.06), post 24.36 (14.87), 6 m 27.29 (18.91) Time effect 3.88 (***p*** **= 0.026**), time*group interaction 0.03 (*p* = 0.967)		
				Coping skills (BRIEF COPE Scale)	People with PD: Pre 47.36 (9.18), post 46.34 (10.28), 6 m 46.58 (12.13) vs pre 47.36 (11.21), post 46.10 (11.39), 6 m 46.28 (11.30) Time effect 0.76 (*p* = 0.471), Time*group interaction 0.01 (*p* = 0.988) Caregivers: Pre 46.41 (10.39), post 48.14 (9.53), 6 m 44.92 (8.18), vs pre 47.68 (10.21), post 49.87 (10.51), 6 m 45.13 (10.82) Time effect 5.95 (***p*** **= 0.004**), time*group interaction 0.25 (*p* = 0.781)		
Yuen, 2020 [16] China	People age 18-80 yrs., with PD. Excluded recent use of antidepressants, recent suicide attempt, history of psychosis, severe comorbidity, H&Y stage ≤4	*n* = 36 Median age: 60 yrs. (intervention) & 65 yrs. (control) H&Y not given.	Conduction Exercise & Self-Accupressure vs usual care + 2 sessions of “health related talk”	Mean +/− SE intervention vs control			Some concerns
				Quality of Life (Chinese PDQ-39)	Pre 43.32(+/−4.75), post 41.32 (+/−5.22), vs pre 40.64(+/−5.31), post 41.07(+/−6.33) Adjusted mean difference between group: −2.25+/− 4.77 (−11.94 to 7.45); *p* = 0.64		
				*Other reported outcomes*: Custom-designed questionnaire: a short form of Non-motor Symptom Scale.			
Van Der Kolk, 2019 [21] The Netherlands	People age 30-75 yrs. with PD H&Y stage I-II, stable medication. Excluded: B-blocking or anti-pscychotic medication, comorbidity that makes them unfit to do the exercises, recent psychiatric disease, dementia, MMSE< 24, unable to perform computer task, no internet at home.	*n* = 130 Mean age 59 yrs. (intervention) & 59 yrs. (control) H&Y 94–95% were stage 2, (range 1–2).	Home-based gamified exercise on a stationary home-trainer vs Active Control: Stretching group Both groups had motivational app.	Intervention vs control: mean (SE) or mean (SE; 95% CI).			Low
				MDS-UPDRS - motor	Pre 29.5 (2.7), post 29.0 (2.5) so change of 1.3 (1.8) vs Pre 27.2 (2.7), post 31.4 (2.5) so change of 5.6 (1.9). Between group difference: −4.2 (1.3; −6.9 to −1.6), ***p*** **= 0.0020**		
				Quality of life (Parkinson’s Disease Questionnaire-39),	Pre 24.9 (2.2), post 26.0 (2.3) so change −0.2 (1.9) vs Pre 24.0 (2.2), post 26.3 (2.3) so change 0.0 (1.9) Between group difference: − 0.2 (1.5; −3.2 to 2.8), *p* = 0.91		
				*Other reported outcomes* Motor scores (Mini-Balance Evaluation Systems test, Timed Up and Go, Six-minute-walk test, pegboard and finger-tapping, fall frequency); Non-motor scales (Hamilton Anxiety and Depression Scale, sleep section of Scales for Outcomes in Parkinson’s disease [SCOPA], Fatigue Severity Scale, gastrointestinal section of the SCOPA Autonomic scale, Montreal Cognitive Assessment, Trial Making Test, Test of Attentional Performance), Cardiovascular fitness (VO_2_ max with graded maximal exercise testing).			
Atterbury 2017 [22] South Africa	People age 50-80 yrs. with PD, H&Y stage I-III. Excluded: MoCA≤17, inadequate functional status, major vestibular, visual, orthopaedic or muscular condition; medication changed n study period.	*N* = 40 Mean Age: 65 yrs. both groups. Mean H&Y: 2.5 (intervention); 2.4 (control), range I-III.	Home based balance exercises on DVD vs Therapist supervised balance exercises	Pre – post mean +/− SD, Home vs therapist groups Between group effect size			High
				Timed Up & Go	Duration Pre 22.96(+/−10.04), post 22.89 (+/− 10.58) [*p* = 0.83] vs pre 19.00 (+/−3.01), post 19.14(+/− 3.29) [*p* = 0.87] Between group treatment effect =0.99		
				*Other reported outcomes*: Functional Gait Analysis; Perceived balance confidence (ABC = Activity Specific Balance Confidence Scale); Intrinsic Motivation Inventory			
Collett, 2017 [18] UK	People with PD *Excluded: Diagnosis of dementia or MMSE < 23, severe depression or psychosis*	*n* = 105 Mean age 67 yrs. H&Y not reported	Self-managed exercise programme vs Self-managed handwriting exercises (control)	Measures listed: delta at 3 months, 6 months & 12 months, then effect size (d) for between groups, considering all 3 follow-up assessments. *Small-moderate effect sizes = 0.1–0.3*			Some concerns
				Motor: 2 min walk	3.8(+/− 3.5); 3.4 (+/− 3.5), 6.7 (+/− 3.6); **d = 0.20** (− 0.44 to 0.45)		
				Health & Wellbeing: EQ5D-5 L SF-36	1(+/− 3); 3(+/− 3); 2(+/− 3); **d = 0.12** (− 0.12 to 0.36) Physical: 1(+/− 3); 1(+/− 3); 4(+/− 4); **d = 0.10** (− 0.14 to 0.34) Mental: 1(+/− 3); 2(+/− 3); 2(+/− 14); d = 0.08 (− 0.16 to 0.32)		
				*Other reported outcomes: Other Motor Measures (MDS-UPDRS-III, 9-hole peg test for dexterity, Timed Up & Go); fitness (VO*_*2*_*, leg power, grip strength); non-motor symptoms (non-motor symptom questionnaire, Fatigue Severity Scale); Health status (BMI, BP, PASE)*			
Collett, 2017 [17] UK *As above: same study, different outcomes*	As above	As above	Self-managed handwriting exercises vs Self-managed exercise programme (control)	Writing (amplitude measures)	[presented as above] Total area (mm^2^): − 6.0 (+/− 4.1), − 2.5 +/− 3.8, − 5.5 (+/− 4.2); **d = 0.32** (− 0.11 to 0.74) % reduction in amplitude: − 10.4 (+/− 7.5), 6.0 (+/− 7.1), − 7.4 (+/− 8.8); **d = 0.11** (− 0.31 to 0.53)		Some concerns
				*Other reported outcomes:* Self-reported writing (MDS-UPDRS- item 2.7)			
Lakshminarayana 2017 [23] UK	People with PD *Excluded: no/limited access to device or internet at home. Dementia or significant cognitive impairment. Major, serious comorbid illness.*	*n* = 215 Mean age 60 yrs.; H&Y not reported	Parkinson’s Tracker App (PTA) vs Control: Treatment as Usual + telephone calls	GLM analysis: difference (95% CI)			High
				Medication Adherence (MMAS-8)	0.39 (0.04, 0.74); ***p*** **= 0.0304** [ANCOVA controlling for age, gender and comorbidity: 0.38 (0.03 to 0.73); *p* = 0.0301]		
				QoL (PDQ − 39)	−0.22 (− 3.95, 3.52); *p* = 0.9102		
				*Other reported outcomes: Quality of Consultations (PCQ-PD); Non-motor symptoms (NMS-Questionnaire, HADS); Beliefs about medication (Beliefs about Medication questionnaire)*			
Sajatovic, 2017 [24] USA	People with PD and depression. *Excluded those unable to walk or high falls risk; and MMSE < 24*	*n* = 30 Mean age 70 yrs.; H&Y range 1–3	Group exercise + chronic disease self-management* vs Self-guided individual exercise + self-guided chronic disease self-management*	“No significant difference” between arms; data not given. Data pooled so results and analysis are pre/post intervention.			High
				Depression (MADRS)	*Pre* mean 21.2 (SD6.3); *Post* 12 weeks 15.2 (8.0) ***p*** **< 0.001**; 24 weeks 14.2 (8.5) ***p*** **< 0.001**		
				*Other reported outcomes*: Self-efficacy (GSE), Cognition (MoCA), Apathy (Apathy Scale), Anxiety (Covi Anxiety scale), Sleep (SCOPA-Sleep), Motor (MDS-UPDRS-III), satisfaction with intervention (custom).			
Advocat, 2016 [25] Australia	People with PD H&Y stage II, age 18-70 yrs., fluent in written & spoken English.	*n* = 72 Mean age 63 yrs. Mean H&Y 2	ESSENCE mindfulness & self-management programme vs Waitlist Control	*Note wait list controls received intervention after the 7 weeks so groups combined for 6 month outcomes.*	7 weeks: change intervention vs control. Effect size, d.	6 months pre-post intervention and control; p for combined group. Effect size, d.	High
				Function & Wellbeing (PDQ39)	−0.54 (−3.41 to 2.32) vs −1.53 (3.64 to 0.57) **ADL domain**: − 2.43 (− 8.11 to 3.25) vs − 2.02 (− 4.66 to 0.62) *p* = 0.89	−0.89 (− 3.71 to 1.93) and − 2.54 (− 6.76 to 1.67), *p* = 0.16. **ADL domain**: − 2.54 (− 6.7 to 1.8) and − 4.17 (− 10.75 to 2.42), ***p*** **= 0.04** (d = 0.23, small)	
				*Other reported outcomes:* Mindfulness (FMI); Mood (Depression Anxiety Stress Scale); Exercise & nutrition (Health Behaviours Questionnaire)			
King, 2015 [26] USA	People with PD plus at least 1 comorbidity; age 40-80 yrs. *Excluded moderate-severe cognitive impairment & those needing assistance with ADLs*	*N* = 58 Mean age 64 yrs. Mean H&Y 2.4.	Sensorimotor-based Agility Boot Camp (exercise programme). 3 delivery methods compared: 1) Home exercise 2) Individual physical therapy 3) Group class	Different pre-post: mean; median (95% CI) for home vs individual vs class			Some concerns
				Physical Performance Test (PPT)	0.71; 0.0 (−0.7, 2.2) *p* = 0.371 vs 1.81; 1.0 (0.69, 2.9) ***p*** **= 0.004** vs 0.55; 0.5 (− 0.4, 1.5) *p* = 0.156. Group comparison: *p* = 0.265 Analysis of effect modifiers: Age *p* = 0.086		
				QoL: PDQ-39	−6.65; −9.0, (− 11.6, − 1.7) ***p*** **= 0.015** vs − 6.30; −5.5 (− 13.1, 0.5) *p* = 0.068 vs − 10.4; − 9.0 (− 16.8, − 4.0) **p = 0.002** Group comparison: *p* = 0.448 Analysis of effect modifiers: Nil significant effects.		
				UPDRS-II (ADL)	ADLs: − 0.65; − 1.0 (2.7, 1.4) *p* = 0.489 vs − 1.67; − 1.0 (− 2.9, − 4.3) ***p*** **= 0.011** vs − 1.90; − 2.0 (− 4.0, − 2.0) *p* = 0.061 Group comparison: *p* = 0.691 Analysis of effect modifiers: UPDRS (*p* = 0.093) and comorbidity (***p*** **= 0.02**)		
				*Other reported outcomes*: Balance: Mini-BESTest; Mobility: TUG, Balance confidence: Activities-Specific Balance Confidence Scale; Apathy: Lille Apathy Rating Scale; UPDRS-III; Self-efficacy (Exercise Self-Efficacy Scale). Potential confounders measured and analysed: Comorbidities, UPDRS, Age, BMI, medication, MoCA, Depression.			
Lawson, 2013 [27] UK	People with PD and anxiety (HADS-A > 8) *Excluded: age of PD onset ≤ 45 yrs; insufficient literacy (WTAR score ≤ 80), Cognitive impairment (ACE-R* score ≤ 83).	*n* = 54 Mean age 66 yrs. Mean H&Y 2.4	Bibliotherapy: “What? Me Worry!?!” online self-help guided resource vs Control: Information only + 1 telephone call	Mean (CI) paired pre-post difference for intervention vs control group. *p* values presented are for pre-post changes; no significant differences found between groups.			Some concerns
				Worry (PSWQ)	− 6.94 (− 13.52 to − 0.37), ***p*** **< 0.05** vs 3.40 (0.52 to 6.28), ***p*** **< 0.05** (note different direction of change)		
				Health status (PDQ-39)	1.0 (− 4.9 to 6.9) vs 2.86 (− 5.88 to 11.60), ns		
				*Other reported outcomes:* Reactions to Uncertainty (IUS); Beliefs about worry (MCQ-30)			
A’Campo, 2010 [28] Netherlands	People with PD + carers. *Excluded: severe psychiatric problems.*	*n* = 64 (PD) + 46 (carers). Mean age 65 yrs. Mean H&Y 2.4 (intervention) & 2.3 (control)	Patient Education Program Parkinson (PEPP) vs Control: Usual Care (delayed intervention)	*Mean difference between intervention arm change (pre-post) and control arm change (pre-post) (95%CI). Bonferoni adjusted significance level of < 0.01.*			Some concerns
				Psychosocial impact of disease (BELA-P-k)	*Patients* Bothered by: 1.74 (− 1.27–4.74), *p* = 0.252; Need for help: 2.04 (− 2.0–6.06), *p* = 0.316 *Carers* Bothered by: 7.05 (2.96–11.14) ***p*** **= 0.001**; Need for help: 11.38 (5.36–17.40) ***p*** **= 0.001**		
				QoL (PDQ-39 for patients; EQ-5D for carers)	*Patients* 4.86 (0.98–8.73), *p* = 0.015 *Carers* Utilities − 0.10 (− 0.24–0.04) *p* = 0.159; VAS − 1.33 (− 11.33–8.66) *p* = 0.788		
				*Other reported outcomes: Depression (Self-rated Depression Scale)*			
^a^Dereli, 2010 [29] Turkey	People with PD, H&Y stage I-III. Excluded: MMSE< 23, disease limiting the ability to perform the exercises, medication changed during study.	*n* = 32 Mean age 67 yrs. Mean H&Y 2	Education + Physiotherapist-supervised exercise vs Education + Self-managed exercise at home.	Mean (SD) pre-post score difference for PT-led groups vs self-managed			Some concerns
				QoL (PDQLQ)	11 (−2 to 23) vs 4 (−16 to 38), ***p*** **= 0.040**		
				Health status (NHP)	−10.5 (−33 to 0) vs −2 (− 13 to 40), ***p*** **= 0.008**		
				UPDRS-II	−3 (−7 to 0) vs −2 (−6 to − 2), ***p*** **= 0.030**		
				*Other reported outcomes:* PD severity (UPDRS); Depression (BDI)			
Tickle-Degnen, 2010 [30] USA	People age ≥ 40 yrs. with PD, H&Y stage II-III. Excluded: MMSE≤26, GDS ≥20, unable to walk without physical assistance, unable to understand and communicate with team, home beyond travel distance to site, medical condition impairing participation	*n* = 117 Mean age 66 yrs. H&Y range 2–3	Self-management rehabilitation programme (2 intensity arms) vs Control: no rehabilitation (medical therapy only)	Quality of Life (PDQ39)	Summary Index adjusted mean (standard error) for 27 h group vs 18 h group vs control; intensity effect, *eta* (95% CI). eta = magnitude of the linear relationship between hours of rehabilitation outcome [interpreted as a product moment correlation (r) effect size.] Post intervention: 27.3 (1.1) vs 27.6 (1.1) vs 31.0 (1.1). *eta* 0.23 (0.05 to 0.40), ***p*** **= 0.01** 2 months follow-up: 28.4 (1.0) vs 28.5 (1.0) vs 30.6 (0.9). *eta* 0.16 (−0.02 to 0.34), ***p*** **= 0.09** 6 months follow-up: 28.2 (1.1) vs 29.2 (1.1) vs 31.5 (1.1). *eta* 0.21 (0.03 to 0.38), ***p*** **= 0.02** ANCOVA: effect of intervention adjusted for baseline F(2,112) = 3.98, ***p*** **= 0.02** Contrast analyses: outcomes co-vary with rehabilitation intensity (group): F(1,112) = 6.48, ***p*** **= 0.01** primarily due to 0-18 h (*p* = 0.03) and 0-27 h (*p* = 0.02) comparisons, not 18-27 h (p = 0.89). Pooled rehab (18 h + 27 h) compared to control: difference 36% (CI 20–53%), ***p*** **< 0.0001**		Some concerns
Grosset, 2007 [31] UK	People with PD *Excluded: significant difficulties using pill bottle.*	*n* = 83 Mean age 64 yrs.; Mean H&Y 2.4	Educational: Verbal & written information vs Control: Usual Care	Intervention arm change (pre-post) vs Control arm change (pre-post)			High
				Medication adherence (MEMS® electronic pill bottles timing adherence)	Median % + 22% vs − 1%, ***p*** **= 0.007**		
				QoL (PDQ-SI)	+ 6 vs + 1.5, p = ns		
				Function (S&E)	−7 vs −3, p = ns		
				*Other reported outcomes: Motor (UPDRS-III)*			
Pearl-Kraus, 2007 [32] USA	People age 30-79 yrs. with PD H&Y stage II-III, able to speak & write in English, able to attend. *Excluded: diagnosed cognitive impairment or MMSE < 23, significant hearing loss, education less than 9th grade.*	*N* = 48 Mean age 68 yrs. H&Y not given	“PD-Collaborative Care” education programme with self-management vs active control: “PD Information Transfer” (education)	Pre, post, mean (+/−SD) Intervention vs control. Repeated measures ANOVA to analyse.			Low
				Quality of Life (PDQ-39)	Pre 24.6 (+/−16.1), post 25.7 (+/− 16.6), 4 weeks 28.0(+/−17.3) vs pre 29.9 (+/− 16.2), post 31.9 (13.4), 4 weeks 28.8(+/−14.6) Pre-post: Group interaction *p* = 0.40, time interaction *p* = 0.31 Post-4 weeks: Group interaction *p* = 0.89, time *p* = 0.12		
				*Other reported outcomes:* Self-efficacy for managing chronic disease (6-item scale)			
Montgomery 1994 [33] USA	People with PD who had applied to enrol on the Propath program.	*N* = 322 Mean age: 68.1 yrs. (intervention); 70.6 yrs. (control) H&Y not given.	Patient education & health promotion vs Waitlist control	Mean change in score (SE) at 6 months for intervention vs control; p* = between group			High
				Questionnaire incorporating questions from UPDRS	Summary score: 0.11 (0.74) [*p* = 0.89] vs 29.7 (0.75) [***p*** **= 0.0001**] P* = 0.007 Patient global assessment: − 0.57 (1.58) [*p* = 0.72] vs 2.92 (1.62) [*p* = 0.075] P* = 0.12		
				Assessment differences in final observations: mean+/−SE; intervention vs control			
				‘Quality of life’ questionnaire	Patient Global assessment: 41.0 (1.8) vs 43.5 (2.0). Self-efficacy (total): 904.0 (24.0) [***p*** **< 0.01**] vs 795.0 (22.0) Spouse stress: 35.0 (1.8) vs 38.2 (1.8) Spouse assessment of participant: 12.1 (0.6) vs 11.3 (0.5)		
				*Other reported outcomes*: Exercise, medication use, health service utilization.			
**Randomised Controlled Trial (Crossover)**							
McNaney, 2019 [34] UK	People with PD H&Y stage I-III with acknowledged daytime drooling problem, able to understand instructions. Excluded: current pharmacological treatment for drooling; insufficient dexterity to use device.	*n* = 27 Median age: 72 yrs. (Immediate) & 75 yrs. (Delayed) H&Y: range II-IV, mean 2.68.	Cueing device for drooling vs Delayed intervention. Treatment as usual in the waiting period.	Pre-post for Immediate Intervention vs Pre-post for Delayed Intervention; Median (IQR) Mann-Whitney U test & Significance of between group difference			High
				ROMP-Saliva	Pre 22 (16–23), post 22 (17–25.5) vs Pre 20 (17–25), post 19 (17–30) U = 83, z = 0.497, *p* = 0.619		
				*Other reported outcomes*: MDS-UPDRS question 2.2; Drooling diary (VAS): Severity, Duration & Frequency			
**Non-randomised Controlled Trials**							
Hellqvist, 2020 [35] Sweden	People with PD & care partners. Excluded: cognitive impairment affecting their ability to understand & respond to outcome measures.	*n* = 92 (PD) + 55 (carer) Mean age, PD: 71 yrs. (intervention), 68 yrs. (control) Mean age carer: 72 yrs. (intervention), 69 yrs. (control). H&Y median 3 (range 1–4) for intervention arm.	Swedish National Parkinson School (NPS) vs matched control: standard care	Median (IQR) pre and post for intervention vs control. Mann–Whitney *U* test for comparisons between groups. Wilcoxon’s signed rank test for within group comparisons.			Mod
				QoL (PDQ-8 for participants with PD)	Pre 28.1 (17.2–39.1), post 23.4 (14.8–37.5) [***p*** **= −0.028**] vs pre 25 (12.5–37.5), post 23.4 (13.3–37.5) [*p* = 0.644] Between group difference: baseline *p* = 0.301, post *p* = 0.713		
				Zarit Burden Interview –short form (care partners)	Pre 7 (3–13), post 8 (3.25–12.75) [*p* = 0.090] vs pre 6 (0.7–12.5), post 5 (2–13.25) [*p* = 0.548]. Between group difference: baseline *p* = 0.495, post *p* = 0.659		
				Health status (EQ-5D)	Pre 0.87 (0.71–0.93), post 0.88 (0.78–0.93) [***p*** **= 0.023**] vs pre 0.86 (0.79–0.93), post 0.86 (0.79–0.91) [*p* = 0.866]. Between group difference: baseline *p* = 0.473, post *p* = 0.279		
				Life Satisfaction (LiSat-11)	“Life as a whole” score: Pre 4 (3–5), post 4.5 (4–5) [*p* = 0.17] vs pre 5 (4–5), post 4.5 (4–5), [***p*** **= 0.011**] Between group difference: baseline ***p*** **= 0.031**, post *p* = 0.868		
				*Outcomes but follow-up results not reported:* Perceived general health (item 1 of the RAND-36) & Function (PADLS). *Other reported outcomes*: Fatigue (PFS-16) Efficacy of self-management education (heiQ)			
Lyons, 2020 [36] USA	People with PD & co-residing partner for ≥1 yr, both willing to enrol. Able to provide informed consent. *Marketed to early PD but later stages not excluded.*	*n* = 39 (PD) + 39 (partners) Age, PD: 71 yrs. intervention& 66 yrs. control Age, partners: 68 yrs. intervention & 66 yrs. control. H&Y not given.	“Strive to Thrive” Dyad Self-management programme vs Waitlist control	Mean (Standard deviation): change in intervention group vs control group; group difference controlling for baseline outcome and age. Cohen’s d [0.2 ~ small, 0.5 ~ medium, 0.8 ~ large].			Mod
				SF-36, Physical Health score	PD: −0.28 (4.69) vs 0.34 (6.33). Group diff −2.50; **d = 0.31** (greater decline in intervention group) Spouses: − 0.86 (5.22) vs − 1.46 (6.27). Group diff − 0.22; d = 0.02		
				SF-36, Depressive Symptoms score	PD: −0.26 (5.20) vs 0.22 (6.09). Group diff − 0.82; d = 0.14 Spouses: − 0.59 (2.90) vs 2.19 (5.68). Group diff − 1.74; **d = 0.29**.		
				Multidimensional Caregiver Strain Index (MCSI)	−0.88 (3.18) vs − 0.45 (2.72) Group diff: − 0.75; d = 0.15		
				*Other reported outcomes*: Measures of self-management behaviours and self-efficacy; Confidence to self-manage (participant and spouse); CES-D scale [Centre for Epidemiologic Studies – Depression Scale]; aerobic activity; Strength based exercise; Mental Relaxation, Illness communication			
Pappa, 2017 [37] USA	People with PD H&Y stage I-III (+carers, but not analysed) *Excluded: suspected dementia or MMSE < 25, psychotic disorder, practical issues limiting participation (*e.g. *lack of transport, non-English speaking).Controls = eligible for study but unable to participate in workshop due to personal circumstances.*	*N* = 46 [+ 6 carers – not in quant analysis] Mean age 68 yrs. Mean H&Y 2.2, range 1–3	Stanford Chronic Disease Self-Management Programme (CDSMP) vs Control: usual care.	Pre, post Intervention vs Pre, post Control, mean (SD)			Mod
				Social Support (ISEL)	Pre 77.8 (5.3), Post 78.4 (5.7) vs Pre 76.6 (4.5) Post 78.2 (4.9) Fs ≤ 1.19, ps ≥ 0.28		
				Other outcome measures given as correlates of ISEL for the intervention group, [outcome results not presented]: Self-Efficacy (CDSES); Health status (PDQ-39); Home, community, socioeconomic & social participation and empowerment (CPI, Involvement in Life Situations Scale, Control over Participation Scale)			
Lun, 2005 [38] Canada	People with PD H&Y stage II-III. Excluded: unstable medication condition, other balance disorder, current regular exercise, health contraindication to exercise, dementia.	*n* = 19 Mean 65 yrs. Mean H&Y 2	Self-managed exercise vs Control: Physiotherapist-supervised exercise	Mean (CI) change pre-post intervention			High
				Motor features (UPDRS-III)	Intervention (home) vs control (physio) group UPDRSm: −5 [***p*** **< 0.022**] vs −5 [***p*** **< 0.009**] Groups pooled for 16 week results: ‘continued exercise’ (CE) vs ‘discontinued’ (DE): UPDRSm: pre 24 (sd = 8), post 15 (sd = 9) vs pre 17 (sd = 5), post 13 (sd = 4)		
				*Other reported outcomes*: BBS, TUG, full UPDRS, ABC			
Lindskov, 2007 [39] Sweden	People with PD. Excluded significant cognitive impairment.	*n* = 48 Mean age: 69 yrs. (intervention), 72 yrs. (control) Median H&Y (range): I (I-III) (intervention) I (I-IV) (control)	Multidisciplinary Education Programme vs Delayed intervention control	Difference (pre-post) in intervention group vs difference in control group, Mean (95% confidence interval) Between group differences evaluated by Mann-Whitney U-Test			Mod
				SF-12 Physical component summary score Mental component summary score	1. (−1.8, 5.8) vs 1.5 (−2, 5.0); *p* = 0.393 2.5 (− 1.0, 5.9) vs 1.1 (− 2.4, 4.6); *p* = 0.361		
				*Other reported outcomes*: Levodopa Equivalents			
**Non-Randomised Non-Controlled: Pre/Post Intervention Designs**							
Li, 2020 [40] Australia	People > 21 yrs. with PD, H&Y stage I-III, comprehend English, live in area & able to attend. Excluded: medically unwell, significant neuropsychiatric disorder (inc cognitive impairment) that precludes consent or participation; unable to mobilize with assistance or did not have a carer to assist if they required assistance.	*n* = 152 Mean age 71 yrs. Mean H&Y = 1.6 (54% = H&Y 1)	PD-Wellbeing programme: Education & Exercise	Pre, post and 1 year follow-up results.			Mod
				Exercise behaviour	Note post-intervention not assessed due to the programme impact on exercise activity. “Exercisers”: Pre: 16%, 1 yr:44% (***p*** **< 0.001**) Exercise less than recommended: Pre: 36%, 1 yr:36% No exercise: Pre: 48%, 1 yr: 19%		
				*Other reported outcomes:* Depression Anxiety and Stress Scale-21 (DASS-21). Univariate analysis for baseline factors and the exercise behaviour outcome performed.			
Mestre, 2020 [41] Canada	People with PD plus care partners. 2 recruitment groups: Newly diagnosed (< 1 yr) and advanced (diagnosis > 8 yrs. or H&Y stage ≥III)	*n* = 100 Newly diagnosed group mean age 69.4 yrs. Advanced group mean age 67.3 yrs. H&Y not given	Integrated Care Network	Difference from baseline (95% confidence interval)			Mod
				Parkinson’s Disease Questionnaire–8 (PDQ-8)	3 months: 1.9 (−0.4 to 4.3); p = 0.08 6 months 2.7 (0.4 to 5.0); ***p*** **= 0.02**		
				MDS-UPDRS: Part II	3 months: 0.3 (−0.6 to 1.2); *p* = 0.49 6 months: − 0.02 (− 0.9 to 0.9); *p* = 0.97		
				Zarit Caregiver Burden Questionnaire.	3 months: 0 (−1.5 to 1.4); *p* = 0.96 6 months: 0.7 (−0.7 to 2.2); *p* = 0.30		
				Perception of support: Patient Assessment of Chronic Illness Case+ (PACIC+)	3 months: 1 (0.9 to 1.2); ***p*** **< 0.0001** 6 months: 1.1 (0.9 to 1.4), ***p*** **< 0.0001**		
				*Other reported outcomes:* Self-management (5As); MDS-UPDRS (parts I & III); Geriatric Depression Score; Program satisfaction (Likert type scale); cost analysis. Clinical Global Impression scales – data not presented.			
Horne, 2019 [42] Australia *Overlap with Li* et al *study sample above*	People > 21 yrs. with PD, H&Y stage I-III, comprehend English, live in area & able to attend. Excluded: medically unwell, significant neuropsychiatric disorder (inc cognitive impairment) that precludes consent or participation; unable to mobilize with assistance or did not have a carer to assist if they required assistance.	*n* = 135 Mean age 71 yrs. Mean H&Y = 1.7 (+/− 0.8)	PD-Wellbeing programme: Education & Exercise	Pre, post (at 6 weeks) and 1 year follow-up results: mean (SD)			Mod
				Physical measures: 2-min walk distance (m) Sit-to-stand (no. in 30s) Timed Up & Go (seconds) Gait velocity (m/s) Berg Balance Scale	Pre 131.9 (41.8), post 151.9 (34.40), [***p*** **= 0.001**]; 12 month (149.5) [***p*** **= 0.001**] Pre 12.49 (3.95), post 15.61 (4.25) [***p*** **= 0.001**]; 12 months 14.88 (4.11) [***p*** **= 0.001**] Pre 10.12 (9.40), post 7.63 (2.91) [***p*** **= 0.001**]; 12 months 7.99 (2.89); [***p*** **= 0.001**] Pre 1.54 (0.44), post 1.74 (0.43), [***p*** **= 0.001**]; 12 months 1.72 (0.43), [***p*** **= 0.001**] Pre 52.2 (7.90), post 54.4 (4.40) [***p*** **= 0.001**], 12 month 54.5 (3.20) [***p*** **= 0.001**]		
				PDQ-39	Pre 34.41 (24.95), post 28.17 (21.82), [***p*** **= 0.001**]; 12 months 29.46 (21.60) [*p* = 0.1]		
				*Other reported outcomes:* DASS-21, PFS-16			
Van Wegen, 2018 [43] Netherlands	People with PD H&Y stage I-III, stooped posture (UPDRS item 28 scores ≥2) that can be actively corrected. Excluded: insufficient cognitive function, relevant comorbidity.	*n* = 15 Mean age 70 yrs.; H&Y not given	“UpRight” posture detection device with feedback *[device inactive but monitoring for pre- phase]*	Posture (Trunk angle measured by device)	Mean (SD) Pre 12.9 (5.9); post 7.5 (5.0); mean change = −5.4 (4.3); ***p*** **< 0.01**		Mod
				*Other reported outcomes*: Satisfaction (custom survey including a VAS); Adverse event (log)			
Hermanns, 2017 [44] USA	People age ≥ 65 yrs. with PD H&Y stage I-IV, able to speak & read English, ambulatory, with written physician approval to engage in the exercise program. Excluded: no access to internet; inability to perform large muscle movements, cognitive impairments that prohibit participation.	*n* = 5 Mean age 73 yrs. H&Y mean 1.7 (range 1–2.5)	Digital Physical Activity Tracker & Online Support Group	PAAI (Physical Activity Assessment Inventory)	Pre 4585, post 2620: % change −42.86		Mod
				Functional Assessment of Cancer Therapy-General (FACT-G)	Pre 440, post 426: % change = −3.18		
				*Other reported outcomes*: Feasibility (useage)			
Esculier 2012 [45] Canada	People with PD; *[healthy people without PD]* MMSE≥24, any comorbidity or limb condition, history of falls.	*N* = 11 *[healthy: n = 9]* Mean age 62 yrs. H&Y not given	Home based balance training *[study compared to paired sample of “healthy” participants – not relevant here]*	Difference (pre-post), median			Low
				Balance: ABC	+ 1 (ns)		
				Mobility: TUG STST Tinetti’s POMA Community Balance & Mobility Scale 10 m walking speed	−1.9 (***p*** **< 0.04**) + 5 (***p*** **< 0.01**) + 4.0 (***p*** **< 0.05**) + 15.0 (***p*** **< 0.02**) −0.7 time to complete (***p*** **< 0.001**)		
				*Other reported outcomes:* Static balance: 1-leg stance duration; programme specific evaluation (likert-type scale)			
Nelson, 2011 [46] USA	Veterans with PD H&Y stage II-III plus spouses with ≥1 chronic medical condition. Excluded: cognitive impairment (MMSE ≤24); depression (CES-D score ≥ 12). Excluded spouses with dementia or depression.	*n* = 13 (PD) + 7 (spouses); Mean age 74 yrs. Mean H&Y 2.5 (2–3)	Stanford Chronic Disease Self-Management Programme (CDSMP)	Quality of Life (PDQ-8)	*Pre* 30.97, *Post* 6 weeks 24.12, 6 months 27.70; p = ns		Mod
				Self-rated health status	*Pre* 2.63, *Post* 6 weeks 2.89, 6 months 2.50; p = ns		
				*Other reported outcomes*: Self-efficacy (SPERC self-efficacy scale); Exercise (SPERC exercise behaviour scale); Pain & Fatigue (SPERC VNS); Depression (CES-D)			
Gruber 2008 [47] Canada	People with PD diagnosed within the last 3 yrs. and H&Y stage 1 or 2.	*N* = 92 Mean age: 52.4 yrs. (site 1) 62.6 yrs. (site 2) H&Y: 97% stages I-II; stage III *n* = 1 (site 1) 96% stages I-II; stage III *n* = 3 (site 2).	Early Management Program (self-management, focussed on exercise)	Pre; post mean (SD)			Mod
				CISM = Chronic Illness Self-Management Questionnaire	Exercise: stretching and/or strengthening: 61.4 (64.2); 91.1 (59.9), ***p*** **≤ 0.001** Exercise: aerobic: 1. (143.3); 145.9 (140.5), *p* = not significant Cognitive symptom management: 1.0 (0.9); 1.5 (0.9); ***p*** **≤ 0.001** Mental stress management/relaxation: 1. (0.5); 1.5 (0.6), ***p*** **≤ 0.01** Communication with physician: 2.0 (1.5); 3.1 (1.3), ***p*** **≤ 0.05**		
				*Other reported outcomes:* Functional Reach; times functional movements; walking speed; Functional Axial Rotation			
Macht, 2007 [48] 7 European countries	People with PD	*n* = 151 Mean 64 yrs.; H&Y mean 2, range 1–5	Patient Education Program Parkinson (PEPP)	Feasibility (intervention evaluation questionnaire)	Range 35–80% average agreement with positive statements and 34–71% average agreement with negative statements.		Mod
				QoL (PDQ-39)	*Pre* mean 30.8 (SD 16.2); *Post* 30.7 (7.7); p = ns		
				Psychosocial impact of PD (BELA-P-k)	*Pre* mean 26.7 (SD 15.6); *Post* 21.0 (14.7); ***p*** **< 0.001**		
				*Other reported outcomes*: Depression (SDS); Mood (VAS)			
Simons, 2006 *Same as Macht above but UK sample* [49] UK	People with PD. Excluded ‘possible cognitive decline’ based on MMSE≤21.	*n* = 36 H&Y 1–4	Patient Education Program: “EduPark” (same as PEPP)	Feasibility (intervention evaluation questionnaire)	Range 40–100% average agreement with positive statements and 0–40% average agreement with negative statements.		Mod
				QoL (PDQ-39)	Data not given. No significant differences were found.		
				Psychosocial impact of PD (BELA-P-k)	Data not given. No significant differences were found.		
				*Other reported outcomes*: Mood (VAS)			
Sunvisson, 2001 [50] Sweden	People with PD H&Y stage I-IV, able to walk independently.	*n* = 43 Mean age 75 yrs. Mean H&Y 1.84	Education programme (information & physical)	Mean (SD) pre, post.			Mod
				UPDRS-II	ADL: 9.48 (5.646), post 9.35 (5.524), [difference 0.140] *p* = 0.7532; 17 weeks post 8.21 (5.655) [difference 1.429] ***p*** **= 0.0098.**		
				Sickness impact profile (SIP)	Pre 11.99 (1.23), post 1.41 (9.52); *p* = 0.0341		
				*Other reported outcomes:* UPDRS-III; Postural Locomotor Manual (PLM) test.			
Jordan, 1993 [51] Australia	Nursing home residents with communication impairments, subgroup presented for those with PD. Coexisting medical problems not excluded.	*n* = 4; Mean age 79 yrs. H&Y not reported	Group communication therapy with self-management strategies	Mean (SD), no p values given			Mod
				Conversation analysis (PCI = Profile of Communicative Interactions)	% Attempted ‘repairs’: Sample 1: pre 89 (11), post 100 (0) Sample 2: pre 100 (0), post 95 (5) % successful repairs: Sample 1: pre 93 (7), post 91.5 (8.5) Sample 2: pre 97 (3), post 100 (0)		
				*Other reported outcomes*: Communication effectiveness (CETI); change of knowledge (custom test)			

*PD* Parkinson’s Disease, *ns* non-significant, *SD* standard deviation, *SE* standard error

* = Experimental & comparator group data pooled for analysis

^a^ = Quasi-randomized: alternate allocation

*Glossary for Measures & Scales*: *ABC* Activities-specific Balance Confidence Scale, *BBS* Berg Balance Scale, *BDI* Beck Depression Inventory, *BELA-P-k* Belastungsfragebogen Parkinson kurzversion, *BMI* Body Mass Index, *BMQ* Beliefs about Medication Questionnaire, *BP* Blood Pressure, *CDSES* Chronic Disease Self-Efficacy Scale, *CES-D* Centre for Epidemiologic Studies Depression Scale, *CETI* Communication Effectiveness Index, *CPI* Community Participation Indicators, *ED5D-5 L* Euro-QOL, *FMI* Freiburg Mindfulness Inventory, *GSE* General Self-Efficacy Scale, *H&Y* Hoehn & Yahr Stage, *HADS* Hospital anxiety and depression scale, *HAM-A* Hamilton Anxiety Rating Scale, *HAM-D* Hamilton Depression Rating Scale, *heiQ* Health Education Impact Questionnaire, *ISEL* The Interpersonal Support Evaluation List, *IUS* Intolerance of Uncertainty Scale, *LiSat-11* 11-item Life Satisfaction Checklist, *MADRS* Montgomery–Åsberg Depression Rating Scale, *MCQ-30* Metacognitions Questionnaire, *MCS* Menta Health Composite Score, *MDS-UPDRS* Movement Disorders Society - Unified Parkinson’s Disease Rating Scale, *MMAS-8* Morisky Medication Adherence Scale, *MoCA* Montreal Cognitive Assessment, *NHP* Nottingham Health Profile, *NMS-Questionnaire* Non-motor Symptom Questionnaire, *PADLS* PD Activities of Daily Living Scale, *PAIS-SR* Psychosocial Adjustment to Illness Scale, *PASE* Physical Activity Scale for the Elderly, *PCQ-PD* Patient-centred questionnaire for Parkinson’s disease, *PDQ-8/39/SI* Parkinson’s Disease Questionnaire (short, full & single index), *PDQLQ* Parkinson’s Disease Quality of Life Questionnaire, *PFS-16* 16-item Parkinson Fatigue Scale, *PSWQ* Penn State Worry Questionnaire, *S&E* Schwab & England, *SCOPA-Sleep* Scales for Outcomes in Parkinson’s Disease – Sleep, *SF-36* Short Form Health Survey, *SPERC* Stanford Patient Education Research Center, *SQLC* Scale of Quality of Life of Caregivers, *TUG* Timed Up & Go Test, *UPDRS III/UPDRSm* Unified Parkinson’s Disease Rating Scale – motor score, *VAS* Visual Analogue Scale, *VNS* Visual Numeric Scale, *WTAR* Wechsler Test of Adult Reading

Details of attrition were not clearly described for all studies. Attrition rates ranged from zero to 50%. For controlled trials, the majority showed greater drop-out rates from intervention arms than control arms, with the exception of one study [49] where the control group drop-out rate was double that of the intervention group. This was thought to be due to disappointment of allocation to the control arm, an issue minimised in other studies through use of waitlist or active control arms.

### Characteristics of selected studies

As summarized in Table 2, a total of 2884 participants, were assessed in studies across USA (10 studies), UK (6 studies), Canada (4 studies), Australia (4 studies), Netherlands (3 studies), Sweden (3 studies), Spain (1 study), Turkey (1 study), China (1 study), South Africa (1 study) and as well as one collaborative European trial (also published with UK data separately). There is overlap between some studies: Collet et al. [40, 48] presented the same sample with different outcomes. Simons et al. [42] present the UK subgroup of the Macht et al. [29] study and personal communication with the authors confirmed overlap between the Li et al. [19] and Horne et al. [20] samples. Nineteen studies were RCTs [21–28, 30–35, 37, 38, 40, 48, 49], one using a crossover design; five were non-randomised controlled trials [36, 39, 43, 45, 47] and the other 12 studies [19, 20, 29, 41, 42, 44, 46, 50–54] were within subject designs with pre- and post-intervention comparisons.

### Characteristics of participants

Seven studies included both people with Parkinson’s disease and their relatives or caregivers [23, 33, 36, 41, 43, 47, 55]. Twelve studies included age as an inclusion criterion. The mean age of participants ranged from 52 years [46] to 79 years [50]. Inclusion criteria specified Hoehn and Yahr (H&Y) stage [56] in half of the studies with the majority restricting to mild-moderate disease and only four studies included H&Y stage IV. Exclusion criteria based on cognition were used by 23 studies; some used a diagnosis of dementia or cognitive impairment, some used a subjective functional interpretation (e.g. cognitive impairment that precludes consent or prohibits participation), and others used cognitive assessment measures, of varying thresholds. Six studies exclusively recruited people with Parkinson’s disease with specific symptoms targeted by the intervention: Depression [22, 25]; anxiety [38]; drooling [24]; posture [44]; and communication difficulties [50]. One study specifically recruited those with another medical condition to analyze of the impact of co-morbidity [31].

### Nature of the self-management interventions

Most interventions were specific to Parkinson’s disease, although two studies examined a more general, established self-management programme, the Stanford Chronic Disease Self-Management Programme (CDSMP), in a sample of people with Parkinson’s disease, and one speech therapy intervention was not condition-specific but tailored to the individual and included a Parkinson’s disease subgroup. The interventions studied were varied but can broadly be divided into five categories as described in Table 3, with topics of content from self-management education and training programmes detailed in Table 4. The interventions are further detailed using the TIDier checklist [57] in Additional file 2.

**Table 3:** Categories of Interventions

Category	Details	Number of Studies
(i) **Self-management education and training programmes**	All but one were group-based, ranging from 4 to 20 participants per group for those that specified, delivered by trained staff, and provided paper hand-outs. The other was delivered remotely, via mail, tailoring according to participant questionnaire responses [33]. Content topics are described in Table 3.	13
(ii) **Self-management training combined with other therapies**.	The other therapies were: • Multidisciplinary rehabilitation • Physical exercise • Cognitive Behaviour Therapy (CBT) • Multidisciplinary care co-ordination • Mindfulness These mostly followed a group session model too, though when combined with CBT and care-coordination this was done on an individual basis. One study compared a group-based delivery of exercise and self-management training with a self-guided equivalent programme [24].	7
(iii) Specific self-management skill of **self-monitoring**	These made use of digital devices to self-monitor: • Symptoms and medication • Posture • Physical activity These were performed on an individual basis, though the physical activity monitoring also included an online peer support group.	3
(iv) Self-management of **individual clinical features** of Parkinson’s	Clinical features targeted: • Anxiety, using CBT - individual • Drooling, via a digital cueing device - individual • Communication difficulties through Speech & Language Therapy (SLT) – small groups, based on diagnosis.	3
(v) Self-management of **specific treatments**, i.e. self-guided treatment programmes	These included: • Medication • Physical exercise • Handwriting exercises • Acupressure & conduction exercise For these the individual was either taught or instructed on how to self-pursue the treatment. These studies either evaluated the self-guided delivery of a treatment, or compared different delivery methods for established interventions.	10

**Table 4:** Content Topics in the Self-Management Education & Training

Topic	Studies	
	Self-management education & training programmes (***n***=13)	Combined with other Therapies (***n***=6)
**Parkinson’s disease** Typically information about the disease and its management.	11 studies [23, 28, 29, 33, 34, 42, 43, 45–47, 54]	4 studies [19, 20, 37, 55]
**Communication and/or Relationships** Social or professional	11 studies [23, 28, 29, 33, 36, 41–43, 45–47]	4 studies [19, 20, 25, 35]
**Psychological Aspects** Stress, anxiety, depression; Coping strategies; future concerns	8 studies [23, 29, 33, 42, 45–47, 54]	6 studies [19, 20, 22, 25, 35, 37]
**Utilising Self-Management Skills**^**a**^ Including self-monitoring, problem solving, information seeking, decision making, maintaining changes, goal setting, action plans.	10 studies [23, 28, 29, 36, 41–43, 46, 47, 54]	3 studies [22, 25, 35]
**Lifestyle** Healthy lifestyle, exercise, diet, sleep hygiene; enriching activities	9 studies [23, 28, 29, 33, 34, 42, 45–47]	5 studies [19, 20, 22, 25, 37]
**Social and/or Financial Support**	8 studies [23, 28, 29, 33, 42, 45, 46, 54]	2 studies [25, 37]
**Function** Activities of daily living, mobility, specific exercises	5 studies [28, 34, 45, 46, 54]	4 studies [19, 20, 25, 35]

^a^Note all interventions incorporated self-management skills, those listed here included specific education sessions on them

### Mode of delivery

A variety of communication and healthcare technologies were utilised for delivery, reducing contact time with professionals. Digital monitoring and cueing devices were employed by four interventions as above, and a further study made us of an electronic pill bottle for collection of medication adherence data [27]. Digital resources demonstrating exercises were used to supplement two interventions: a mindfulness CD [37] and physical exercise DVD [26]. Remotely delivered CBT based interventions were evaluated in two studies [22, 38]. Two studies provided home exercise equipment with game components to engage and motivate participants [32, 51].

### Duration and intensity of the interventions

The interventions varied in intensity and duration. One included only a single one-off session [27]; all others involved repeated sessions, typically regular weekly sessions with intensity ranging from 1 h per week to 3 h twice a week. Some self-directed interventions involved a recommendation to participate daily. Two studies used fixed intervention points over a longer time period: 3–4 sessions over 6 months [34, 53]. Aside from the one-off session intervention, the lengths ranged from 2 weeks to 6 months.

### Study outcomes

Half of the studies assessed outcome immediately following the intervention and the other half also included a delayed follow-up to examine sustainability of outcomes. Whilst frequent, the QoL measures were often not the primary outcomes and as such, the RCTs were not necessarily powered specifically to detect a change on this measure. The majority of studies used primary outcome measures related to the specific clinical issue targeted by the intervention. Participant evaluation of the intervention was included in 15 studies, discussed in Additional file 4.

### Effect of interventions

#### Self-management education and training programmes

Three RCTs evaluated group Parkinson’s disease-specific education programmes that include *in-person training in self-management skills*. None showed significant improvements in QoL compared to controls. One [23], reported significantly increased psychosocial adjustment in caregivers in the intervention compared to the usual care control group. There was also a trend towards improvement in QoL for participants with Parkinson’s disease for the intervention group and deterioration in the control group, but after Bonferroni correction the difference was not statistically significant. Another shorter intervention (3 sessions) [28], showed no significant effect pre- versus post intervention or compared to a control group who received information only. The third found improvement in PDQ-39 scores, psychosocial adjustment and caregiver ‘coping’, in both intervention and control groups, with the latter receiving multidisciplinary education without the psychological components, but no significant group effect [33]. Another RCT investigated a related intervention: an individualised education-based intervention, delivered to the participant by mail [34]. This showed improvements in the intervention compared to the usual care control arm in the Parkinson’s Questionnaire outcome measure which included functional items modified from the UPRDS. Score for the self-efficacy subdomain of their custom QoL questionnaire were also significantly better in the intervention than control group at follow-up, but not the total score, and there was no baseline measurement of this questionnaire to evaluate change following the intervention.

Three non-randomised controlled trials evaluated similar self-management interventions [43, 45, 47], one of which showed improvements pre-post intervention in QoL and health status (PDQ-8, EQ5D and LiSat-11) [47], not replicated in the other two. Another of these did show a small improvement in caregiver strain in the intervention arm compared to control, but alongside greater deterioration in physical health for the people with Parkinson’s disease and greater deterioration in caregiver depressive symptoms in the intervention versus control [43]. It also showed improvements in self-management outcomes that were greater in the intervention arm than control, particularly for caregivers.

Another non-randomised controlled trial evaluated the well-established Stanford CDSMP (not Parkinson’s disease -specific), but did not present the QoL results, instead using it as a factor in analysis [36]. The primary outcome was a social support measure which did not significantly change.

A further five studies, of which two overlap in samples, were non-controlled trials using pre-post intervention evaluation [29, 41, 42, 46, 54] . Regarding our outcomes of interest, one demonstrated significant improvements in psychosocial adjustment for the participants with Parkinson’s disease [29] and another showed improvements in activities of daily living at the delayed follow-up assessment (17 weeks). Another, that did not include our outcomes of interest did show improvements in self-management outcomes and a physical measure of axial rotation [46]. No other positive findings were reported.

#### Self-management training combined with other  therapies

Two of the four RCTs in this category present positive findings. A larger RCT (*n* = 117) evaluated MDT rehabilitation combined with self-management training, that focused predominantly on day-to-day tasks. They report significantly improved QoL (PDQ-39, primary outcome) compared to controls who received no rehabilitation [35]. Findings were sustained, albeit lessened over time (6-month follow-up). Additionally an RCT evaluated an intervention delivering cognitive behaviour therapy (CBT) with self-management training by telephone. This showed significant improvements in both symptoms of depression, the primary outcome that it was targeted to address, and QoL (indicated by the Mental Health composite Score of the SF-36), compared to those in the control arm [22].

Another RCT evaluated a group-delivered course that combined mindfulness and self-management training. In pre-post intervention comparison for all participants (intervention and waitlist controls) after 6 month, significant improvement in the ‘ADL’ domain of the QoL measure was seen [37]. However, improvements in QoL were not significant compared to waitlist controls. The fourth randomized trial evaluated self-management training combined with exercise for people with Parkinson’s disease and depression, comparing group based and self-guided delivery, but without a no-intervention control [25]. No difference was found between the groups. When data was pooled across both arms, there was a significant improvement in depressive symptoms pre/post intervention. QoL measures were not used.

Two non-controlled studies, with some sample overlap between them, evaluated a programme that combined multidisciplinary education with physical exercise, emphasizing self-management. They demonstrated improved mobility and balance outcomes, as well as improved QoL at short-term follow-up. The QoL improvement was not sustained at not long-term follow-up 12 months later [20], though exercise behavior was [19]. A non-controlled study of an integrated care model, incorporating multidisciplinary professionals and emphasizing self-management, reported significant improvements in QoL at 6 months, but not 3 months [53].

#### Self-monitoring interventions

An RCT evaluating a ‘Parkinson’s tracker app’, did not show statistically significant improvements in QoL compared to the control. There was significant improvements compared to controls in the primary outcome of self-reported medication adherence [30] and improved perceived quality of Parkinson’s disease care.

The other trials were small, non-controlled feasibility studies of narrow clinical focus: A study of a physical activity tracker plus online support group for older people with Parkinson’s disease [52] and a study of an ‘ambulatory posture detection device’ [44]. The former did not show any significant improvements, including QoL or function. The latter showed significant improvement in trunk angle as a measure of stoop (primary outcome) but it did not use QoL or function measures.

#### Self-management of individual clinical features of Parkinson’s disease

One RCT evaluated a CBT-based self-help resource with telephone support against information only with one telephone call [38]. Pre-post intervention comparison showed a significant reduction in worry and intolerance of uncertainty, which the intervention was targeted to address, whilst worry significantly increased in the control group, but the between-group difference was not statistically significant. There was no significant difference pre-post intervention or group difference for QoL (PDQ-39).

The other studies in this category were small and did not evaluate QoL or wellbeing outcomes, but rather measures of the targeted feature. One was a small pilot RCT (cross-over) (*n* = 27) evaluating a digital cueing device for drooling. The validated measures of drooling symptoms showed no significant improvement pre-post or between groups, but the improvement in ‘overall severity’ domain of a self-reported symptom measure using a visual analogue scale was significantly better than the control. The other was a pre-post intervention evaluation of speech and language therapy for nursing home residents with communication difficulties [50]. One of the four participants with Parkinson’s disease was seen to improve on a communication effectiveness measure and two on a knowledge measure, but not the other participants.

#### Self-guided treatment programmes

A variety of treatments were studied: exercise; medication management; and acupressure & conduction therapy.

An RCT evaluated a physiotherapist-supported, self-guided exercise programme compared to a self-guided handwriting exercise control group [40, 48]. The exercise group showed significant improvements in QoL and wellbeing (EQ5D-5 L and SF-36), compared to the handwriting control, though the effect sizes were small. Significant improvements in the exercise group were seen in the MDS-UPDRS motor scores (moderate effect size) [40] and handwriting scores in the handwriting group (small effect size) [48]. An RCT evaluating a home based aerobic exercise programme demonstrated significantly improved motor scores, the primary outcome measure, compared to a control group undertaking stretching exercises [32]. Improvements in QoL (PDQ-39) were not significantly different between the groups. A trial of an exercise intervention comparing different modes of delivery, discussed in 4.10 below, showed improvements in QoL (PDQLQ) but had no control group [21]. A pre-post intervention comparison in a pilot study of home-based balance training did not use QoL or wellbeing outcomes. It showed significantly improved mobility, but not balance [51].

An RCT investigated a single educational session regarding pharmacology of Parkinson’s disease treatment, delivered one-to-one to the participants in the outpatient setting by a clinician [27], aiming to improve medication adherence. No significant changes were seen for QoL or function measures. Significant improvements were seen in the primary outcome of medication adherence, measured using electronic pill bottles, compared to the control arm.

A pilot RCT, which evaluated self-administered acupressure and conduction therapy, did not find significant improvements in QoL (PDQ-39, Chinese version), the primary outcome measure, compared to the control arm [49]. This study had a high attrition rate of 50% in the control arm (24% in intervention arm).

### Comparisons of delivery methods

The one study using QoL as a primary outcome measure, significantly favoured the physiotherapy-supervised exercise group over the self-guided exercise group [21]. Similarly, improvements in health status and UPDRS parts I-III (separately and total) were significantly greater in the physiotherapist-supervised group. Both groups had also received individualised education about Parkinson’s disease and the exercise programme.

The other studies, using motor, mobility or physical performance outcomes, did not show significant differences between therapist-led and self-guided exercise groups [26, 31, 39]. Only one of these included QoL and function measures. There were improvements in physical performance and ADLs in the *individual* physiotherapy arm but not the self-guided or group therapy arms, and improvements in QoL in the self-guided and group therapy arms but not the individual physiotherapy arm [31], the group differences were not significant.

### Components of interventions

Analysing the interventions using the PRISMS Self-Management taxonomy, it is clear that most interventions are complex, multi-component, targeting different aspects of self-management. Table 5 illustrates the self-management components of the interventions that were associated with improvements in QoL, wellbeing or function, either compared to controls (4 studies – indicated by *) or pre-post intervention evaluation (9 studies). Table 6 shows the components of the interventions that did *not* demonstrate improvements in these outcomes. Interventions that were reported to be effective included different combinations of components. However, components that appear more frequently in interventions resulting in improvement than in those that do not are: information about resources; training or rehearsing psychological strategies; social support; and lifestyle advice and support.

**Table 5:** Intervention Components (PRISMS Taxonomy) in studies with significant improvements in Quality of Life, wellbeing or function

Intervention	Patient Education Programme Parkinson’s	Self-guided Exercise	Rehab + Self-management	Telephone- Cognitive behaviour therapy	Psycho-education	Propath	National Parkinsons School	Education	Early Management Programme	Essence Mindfulness	PD Wellbeing Programme	Integrated Parkinson’s Care Network	Home Exercise
**Study significant finding** *significantly better than *control* Others are pre-post changes m=month, w=week	*improved psychosocial impact of disease on carers (A’Campo) [28] Improved psychosocial impact of disease (Macht) [48]	*improved health & wellbeing (Collett) [18]	*improved QoL (Tickle-Degnen) [30]	*improved health status (Dobkin) [19]	improved QoL, psychological adjustment & carer coping (Navarta-Sanchez) [20]	Improved UPDRS & self efficacy domain of QoL measure (Montgomery) [33]	improved QoL & health status (Hellqvist) [58]	improve (ed ADLs (17w not post) (Sunvisson) [50]	improved SM (Gruber) [47]	improved QoL ADL domain (Advocat) [25]	improved QoL (post, not at 12 m) (Horne) [42]	improved QoL (6 m, not 3 m), perception of support (3+ 6 m) (Mestre) [41]	improved QoL (King) [26]
1. Information about Parkinson’s/ its management	✓	x	x	✓	✓	✓	✓	✓	✓	✓	✓	✓	x
2. Information about available resources	✓	x	x	✓	✓	x	✓	x	✓	x	✓	✓	x
3. Clinical action plans	x	Exercise plan	✓	✓	x	✓	✓	x	✓	x	x	✓	Exercise plan
4. Regular clinical review	x	Therapist, short term	Therapist, short term	Therapist, short term	x	Remote, short term	x	x	x	x	Therapist, short term	✓	x
5. Monitoring of condition with feedback	✓	Therapist-led	✓	x	x	Questionnaire	✓	Self-monitor, no feedback	x	Self-monitor, no feedback	Self-monitor, no feedback	✓	x
6. Practical support with adherence	x	x	x	✓	x	x	x	x	x	x	✓	x	x
7. Provision of equipment	x	Gym access	x	x	x	x	x	x	x	CD pack	x	x	x
8. Access to advice or support	x	✓	x	x	x	x	x	x	x	x	x	✓	x
9. Training to communicate with HCPs	✓	x	x	✓	x	x	✓	x	✓	Information about professional support	x	x	x
10. Training for everyday activities	x	exercise	✓	x	x	x	x	✓	✓	x	x	indirect	exercise
11. Training for practical self-management activities	x	✓	✓	✓	✓	x	✓	✓	✓	✓	✓	Indirect	x
12. Training for psychological strategies	✓	x	✓	✓	✓	x	✓	✓	✓	✓	x	Indirect	x
13. Social support	✓	x	Group based; ✓18 h group	✓	✓	x	✓	Group based	Group based	✓	✓	Indirect	x
14. Lifestyle advice and support	✓	x	✓	✓	✓	✓	✓	✓	✓	✓	✓	✓	x

Description where there is partial or ambiguous inclusion of component

*HCP* Healthcare professional

x = not present in intervention

✓= present in intervention

**Table 6:** Intervention Components (PRISMS Taxonomy) in studies without significant QoL and wellbeing changes

Intervention/ Study	Medication education (Grosset) [31]	Parkinson’s Tracker App (Lakshminarayana) [23]	CBT guided reading (Lawson) [27]	Education + Home exercise (Dereli) [29]	Home exercise + motivation (Van der Kolk) [21]	Self-acupressure & conduction therapy (Yuen) [16]	PD-Collaborative Care (Pearl-Kraus) [32]	Strive to Thrive (Lyons) [36]	Education Programme (Lindskov) [39]	Chronic Disease Self-Management Programme (Nelson) [46]
1. Information about Parkinson’s disease/ its management	✓	✓	✓	✓	x	x	✓	✓	✓	x
2. Information about available resources	x	x	x	x	x	x	✓	x	✓	x
3. Clinical action plans	x	x	x	x	✓	x	✓	✓	x	✓
4. Regular clinical review	x	x	x	Therapist, short term	x	x	x	x	x	x
5. Monitoring of condition with feedback	x	✓	“discuss progress”	x	✓	x	x	Self-monitor, no feedback	x	Self-monitor, no feedback
6. Practical support with adherence	x	✓	✓	✓	✓	x	x	x	x	x
7. Provision of equipment	x	app	x	x	Exercise equipment + app	x	✓	x	x	x
8. Access to advice or support	x	x	x	x	x	x	x	x	x	x
9. Training to communicate with HCPs	x	✓	x	x	x	x	✓	✓	x	✓
10. Training for everyday activities	x	x	x	exercise	exercise	x	✓	x	✓	x
11. Training for practical self-management activities	x	x	x	✓	✓	✓	✓	✓	✓	✓
12. Training for psychological strategies	x	x	✓	x	x	✓	x	✓	✓	✓
13. Social support	x	x	x	x	Support messages	x	✓	✓	✓	✓
14. Lifestyle advice and support	x	x	x	x	x	x	✓	✓	✓	✓

X = not present in intervention

✓= present in intervention

Intervention evaluations by participants (questionnaires and/or interviews) do not identify clinically effective components but offer insight to well-received components. One report identified the topic of ‘stress management’ as the most highly rated session. Six evaluations specifically highlighted social or peer support aspects, such as sharing of experiences, as being particularly beneficial [25, 29, 36, 37, 41, 59]. Evaluation of the physical activity tracker with online support group identified peer support as a mechanism for behaviour change [52].

One of the four positive RCTs included caregiver participation in the intervention, finding positive impacts on caregiver outcomes. Four of the 10 studies showing improvements in QoL or function following the intervention for the person with Parkinson’s disease, compared to two of the 10 that did not find such improvement, included caregivers in the intervention.

## Analysis

**Fig. 2:**
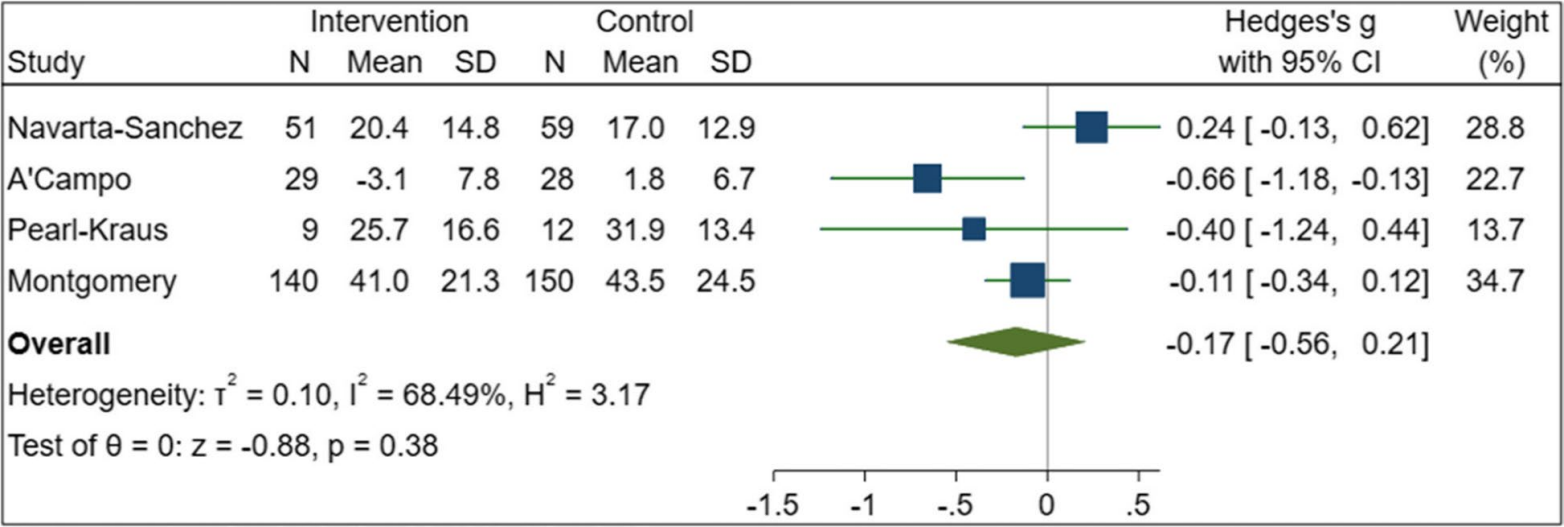
Forest plot for four RCTs evaluating group-based self-management education and training programmes effect on QoL against standard care

The heterogeneity of study designs, interventions and outcome measures allowed for pooling of data from only four category (i) studies. Meta-analysis, as illustrated in Fig. 2, shows the pooled data for QoL outcomes for four RCTs of *self-management education and training programmes.* The risk of bias in these studies ranged from low to high. The three group-based programmes all used the PDQ39 outcome measure, although only one provided data for change in scores. The fourth study, of an individual mail-out education intervention, used a QoL custom questionnaire at follow-up only. Pooling the results, the standardised mean difference (Hedges g) of − 0.17 (− 0.56, 0.21) suggests a possible small benefit from interventions, but there is no statistical evidence to confirm this (*p* = 0.38). The I^2^ value of 68% suggests a relatively high level of heterogeneity between the studies. By the GRADE approach, the certainty of the evidence is deemed “very low” (for the Evidence Profile, see Additional file 5).

Three non-randomised trials of category (i) interventions with similar outcomes were not suitable for inclusion due to baseline imbalance in scores, which was adjusted for in the papers but did not provide insufficient information to adjust for in meta-analysis. Interventions in other categories could not be synthesised quantitatively as they used different therapies and targeted different clinical aspects of Parkinson’s disease.

## Discussion

### Effectiveness of interventions

Overall, there is a paucity of studies of high quality randomised controlled trials of self-management in Parkinson’s disease. Most of the identified studies are small and of low quality. The variable applications of self-management concepts confer heterogeneity of study aims, content and outcome measures. This is consistent with findings of the previous review of self-management interventions in Parkinson’s disease [9], although even more apparent in our review given the broader definition used.

Meta-analysis of the few interventions with similar content, target and outcomes did not show a significant effect on QoL compared to the control group for self-management education and training programmes. Amongst the other studies using a control group, significant improvements in QoL were reported with self-management training with CBT [22], MDT rehabilitation [35], and self-guided exercise [40]. However, across the whole selection of controlled studies identified, this was not a consistent finding. Many studies did not use QoL, wellbeing or functional outcomes as primary outcomes which may account for some of the negative results. One study did show significant improvements in perceived psychosocial impact of disease for caregivers [23]. 

Comparisons of baseline to post intervention assessments did suggest that self-management interventions and self-guided treatments may have positive effects on these outcomes, including for some group-based education and self-management programmes [29, 46, 47, 54]; a remotely delivered individual education and self-management programme [34]; interventions combining education and self-management training with exercise [20, 31] or mindfulness [37], and an integrated care model combining multidisciplinary professional input, education and self-management training [53]. Further controlled studies on these interventions are however required.

### Intervention components

Limited conclusions about specific intervention components can be drawn due to the low quality and heterogeneity of evidence. There is a suggestion that inclusion of a greater number of components, addressing a range of aspects of self-management, may be beneficial, which is consistent with past research for self-management in long term conditions [7]. The majority of interventions incorporated information about Parkinson’s disease and its management, but not all found significant improvement in outcome. Information alone does therefore not appear sufficient to improve QoL, in keeping with research in other conditions [7]. Components more common of interventions showing improvements (typically pre-post rather than compared to control), but less common in interventions not showing improvement, were: *information about resources*; *training or rehearsing psychological strategies*; *social support*; and *lifestyle advice and support*. Whilst not a specific component of self-management, exercise was emphasised in many of these interventions, which is well recognised to be important in Parkinson’s disease [60]. There is notable overlap between these components and those identified as important through our previous synthesis of qualitative literature on self-management in Parkinson’s disease [14]: (1) *medication management*, (2) *physical exercise*, (3) *self-monitoring*, (4) *psychological strategies*, (5) *maintaining independence*, (6) *social engagement*, and (7) *knowledge and information*.

### Other intervention variables

The intensity and duration of interventions may play a role in effectiveness, which in clinical practice would need to be balanced with cost and resource considerations. There is no predictable association between these factors alone and the effect of interventions discernible in this review. A study that appears to have similar content and components to those showing pre-post improvements, but did not itself find significant improvements, was notably shorter than average, with only 3 sessions [28].

Two of the four positive RCTs, and seven of the 10 studies conferring pre-post intervention improvements were group-based programmes. Others used remote methods of delivery (two self-guided following instructions, one telephone therapy, and one postal guidance), supporting this possibility for future interventions. Other than self-monitoring devices, one of which incorporated information about Parkinson’s disease from charities onto an app, no study used a digital package to support self-management at home. Past research in long term conditions more generally has not elucidated a particular education delivery method as being more favourable than others [7].

Previous research found support of family to be associated with better self-efficacy which was predictive of better self-management in Parkinson’s disease [61]. Involvement of caregivers within self-management interventions has been postulated as a mediator of effectiveness [9]. Whilst our findings suggest a trend to support this, the evidence is not conclusive.

Most attrition in the studies was labelled “lost to follow-up” or “medical reasons”, without detailed reasons. Some reported practical and logistical reasons, such as transport, time and cost. Two studies report individual drop-outs based on the group nature of the intervention – one discouraged by seeing another participant’s condition [41], and one put off by the expectation to talk with others about their condition [28]. These factors may influence design of future interventions.

### Strengths and limitations

The main strength of this review is the robust and reproducible methodology, adhering to PRISMA guidelines, and the broad inclusion criteria to provide a comprehensive review of studies in the field. The methodology involved multiple databases, extensive search terms, and two independent reviewers.

Self-management is an inherently broad concept, further evidenced by the diversity of interventions described in this review. Other interventions may incorporate self-management aspects, for example singing or dance interventions, but without explicit self-management conceptualisation. Thus, some studies may not have been identified due to lack of attribution or recognition of self-management concepts. Use of resources like the TIDier checklist to describe interventions, and the PRISMS Taxonomy of Self-Management will help alleviate this issue in the future. Furthermore, most studies lacked detailed description of the control arm, particularly regarding ‘usual care’. Since this may include some self-management components, such as ‘provision of equipment’, this limits the interpretation of active intervention components and their effectiveness.

Non-English language articles were excluded which excluded 3 potentially relevant studies: one of a self-management programme in Parkinson’s disease [58] (Korean), one of self-catheterisation for urinary symptoms in Parkinson’s disease [62] (French), and one of group physiotherapy [63] (German) for which the relevance could not be determined from information available.

Overall, the risk of bias in the studies, in relation to QoL and wellbeing outcomes, was high. The main determinant of this was lack of blinding along with self-reported outcome measures, which are inherent to the nature of these interventions.

## Conclusion

Despite the increasing interest in the topic of self-management in Parkinson’s disease, there are insufficient high quality RCTs in this field to draw firm conclusions on the effectiveness of self-management interventions in this population.

No single component was consistently associated with the success of self-management interventions to improve QoL, wellbeing or function. Whilst the previously recognised key components of education, goal setting, and problem solving were common, they did not distinguish effectiveness. Components that were more common in interventions associated with improvements in these outcomes, albeit often not compared to controls, were: *information about resources*; *training or rehearsing psychological strategies*; *social support*; and *lifestyle advice and support*. Focused interventions relating to specific self-management skills or self-management of specific clinical features or treatments do show promise for improving the targeted feature, but the significance of this for the individual remains to be shown.

More high quality RCTs are needed to determine the clinical effectiveness of self-management in PD, with suitable carefully chosen clinically relevant outcomes.

## Supplementary Information

**Additional file 1.** Search Terms.

**Additional file 2.** Intervention Details – TIDieR [54].

**Additional file 3.** Risk of Bias Assessment.

**Additional file 4.** Results of Intervention Evaluations.

**Additional file 5.** GRADE Approach: Evidence Profile for Meta-analysis.

## Data Availability

The search strategies used in this systematic review are available in the supplement. All data used in this systematic review are from previously reported studies and datasets, which have been cited.
