# Supplementary material for: Systematic review and meta-analysis of clinical effectiveness of self-management interventions in Parkinson’s disease
Source: BMC Geriatr. 2022 Jan 11;22:45. doi: 10.1186/s12877-021-02656-2 (PMC8753859; doi:10.1186/s12877-021-02656-2)
Supplement: Supplementary file 1 — Additional file 1. Search Terms. [file 12877_2021_2656_MOESM1_ESM.docx]

**ADDITIONAL FILE 1: Search Terms**

***MEDLINE (via Ovid):***

1. exp PARKINSON DISEASE/

2. parkinson*.mp. [mp=title, abstract, original title, name of substance word, subject heading word, floating sub-heading word, keyword heading word, protocol supplementary concept word, rare disease supplementary concept word, unique identifier, synonyms]

3. exp Self Care/ or exp Self-Management/

4. self-management.mp. [mp=title, abstract, original title, name of substance word, subject heading word, floating sub-heading word, keyword heading word, protocol supplementary concept word, rare disease supplementary concept word, unique identifier, synonyms]

5. self care.mp. [mp=title, abstract, original title, name of substance word, subject heading word, floating sub-heading word, keyword heading word, protocol supplementary concept word, rare disease supplementary concept word, unique identifier, synonyms]

6. 1 or 2

7. 3 or 4 or 5

8. 6 and 7

***Embase (via Ovid)***

1. exp Parkinson disease/

2. parkinson*.mp. [mp=title, abstract, heading word, drug trade name, original title, device manufacturer, drug manufacturer, device trade name, keyword, floating subheading word, candidate term word]

3. self-management.mp. or exp self care/

4. self care.mp. [mp=title, abstract, heading word, drug trade name, original title, device manufacturer, drug manufacturer, device trade name, keyword, floating subheading word, candidate term word]

5. 1 or 2

6. 3 or 4

7. 5 and 6

***PsycINFO (via Ovid)***

1. exp PARKINSON'S DISEASE/ or parkinson*.mp.

2. self-management.mp. or exp Self-Management/

3. 1 and 2

**Web of Science**

PARKINSON* and self-management
