# Supplementary material for: Systematic review and meta-analysis of clinical effectiveness of self-management interventions in Parkinson’s disease
Source: BMC Geriatr. 2022 Jan 11;22:45. doi: 10.1186/s12877-021-02656-2 (PMC8753859; doi:10.1186/s12877-021-02656-2)
Supplement: Supplementary file 2 — Additional file 2. Intervention Details – TIDieR [54]. [file 12877_2021_2656_MOESM2_ESM.docx]

**ADDITIONAL FILE 2: Intervention Details – TIDieR**[54]

| **Item 1**  **Intervention** | **Item 2**  **Rationale** | **Items 3-7**   - **Materials** - **Procedures** - **Who** - **Mode of Delivery** - **Location & Infrastructure** | **Item 8**  **When & How Much** | **Items 9-12**  **Tailoring, Modifications, Planned & Actual Adherence & Fidelity Assessment** |
| --- | --- | --- | --- | --- |
| 1. **Self-management education and training programmes** | | | | |
| **Patient Education Program Parkinson (PEPP)** A’Campo, Macht  [20, 23]  **EduPark,** Simons [19] | To improve knowledge and skills related to self-monitoring, health promotion, stress management, depression, anxiety, social competence and social support.  *Studying feasibility* | Manual-based; sessions: information (general information, symptoms of PD, involving professionals), self-monitoring, health promotion & empowerment, stress management, management of anxiety & depression (patients) / caregiver’s challenge (carers), social competence, social support, evaluation.  Small group meetings: 5-7 people per group  Delivered by trainers, who received 2-day intervention training  Patients & caregivers separately but simultaneously  Session structure: introduction, active information, exercise, homework, appetizer. 15minute break.  *Location of intervention delivery not stated.* | 8 x weekly 90 mins sessions  *Both studies assessed at the start and end of the programme.* | Delivery standard but discussions would depend on the participants’ specific contributions. “Session content is adapted to suit the needs of either the PD participants or the carer participants.”  No reported modifications. Monitored by attendance records. |
| **Psycho-education** Navarta-Sanchez [32] | To strengthen quality of life, psychosocial adjustment, and coping – for people with PD and their informal caregivers. | Manualized programme. Psychological component: Promotion of coping and psychosocial adjustment - delivered in 4 sessions, led by a psychologist, GP and an expert patient. Included everyday life, positive self-esteem, empathy & patience, stress management, information seeking, living in the present, partaking in activities, and normalization. Encouraged to reflect on and share personal experiences and coping.  Information components: general information about PD, healthy lifestyles and community resources. Education delivered by: GP, neurologist, nurse, social worker.  Delivered at the participants Primary Care Centre. Patient & Caregivers received the session at the same time in a different room. In groups of up to 15-20.  *Control: 5-week education programme (information components) delivered in the same way.* | 90minute sessions per week for 9-weeks.  *Study follow-up after intervention + at 6months* | Delivery standard but discussions would depend on the participants’ specific contributions.  No reported modifications. Monitored by attendance records. |
| **National Parkinson’s School (NPS)**  Hellqvist [41] | To provide knowledge and tools to enhance their ability to live and handle life with PD | Based on PEPP (above) with self-monitoring and self-management components.  Small group sessions, including care partners.  Qualified instructor: a health care professional experienced with PD and trained to deliver the programme.  Specific topic introduced by the instructor, then group discussions, closing with a 15-minute relaxation exercise. Application though practical exercises and homework assignments.  Topics: Introduction, Self-monitoring, Stress, Anxiety & Depression, Communication, Enriching Activities, My Future life with PD.  *NPS already implemented in clinical care and provided at participating clinics.* | Seven, two-hour sessions  *Assessed at the start and end of the programme.* | Standardised programme but with discussion & application tailored to the individual.  No reported modifications. Monitored by attendance records. |
| **Education programme,** Lindskov [42] | To improve patient reported health outcomes. | Standardized teaching programme delivered by healthcare professionals with teaching experience: physician, nurse, occupational therapist, physiotherapist, dietician, speech therapist, psychologist, social worker and dental hygienist.  Sessions included: information on PD and its management, daily living, mobility, diet & nutrition, coping strategies, communication, oral hygiene and related symptoms, financial & social support. Conducted in the outpatients.  1^st^ hour – information delivered in interactive dialogue. Discussion & sharing of personal experiences encouraged.  2^nd^ hour – exercises demonstrated to people with PD, family members took part in a nurse-led peer support group. | Weekly 2-hour sessions for 6 weeks  *Assessed at the start and weeks after the end of the programme.* | Standardised programme but with discussion depends on participants.  No reported modifications. Monitored by attendance records + a nurse participated in every session for standardization and continuity. |
| **“Strive to Thrive” self-management programme,** Lyons [43] | To improve physical health, depressive symptoms and carer strain as well as better self-management behaviours, increased exercise and improved communication within-couple. | Structured train-the-trainer model: programme leaders were peer trainees trained by the investigator who had completed the 4-day training session at Stanford University.  Group of people with PD and/or spouses.  Based on CDSMP [above - includes aspects of chronic conditions and teaches self-management skills] with an extra week added for PD-specific content.  Location not stated. | 7 weekly sessions, duration of sessions not stated.  *Assessed at the start and end of the programme.* | Followed a structured programme for fidelity. A trained leader (program co-ordinator) also observed at least 2 sessions for each cohort of workshops. No reported modifications. Monitored by attendance records and the observer. |
| **Education programme**  Sunvisson [49] | To guide understanding of how 4 domains (psychological, biological, sociological & physiological) interact in their lives and manage sickness-related difficulties in daily life. | Outpatient programme based on the structure of connection model. Each session: 1hour of dialogue, 1hour of physical performance.  Taught by a nurse and a physiotherapist.  Information topics: physical & psychological symptoms of the disease, dialectical liaison between body & mind, medical treatments & side effects, influences from physical surrounds & social networks, obtaining and maintaining good self-care. Participants were asked to complete some home-based preparatory tasks. Handout with figure explaining the “structure of connection” model provided.  Physical training: coordination, balance, body rhythm, stretching, relaxation & body language. Taught to initiate movements.  Information and physical sessions were dependent on each other. | Two weekly 2-hour sessions for 5weeks.  *Assessed at the start and end of the programme + 3months follow-up* | Time used for the various topics varied according to interest. |
| **PD-Collaborative Care**  =education with self-management  Pearl-Kraus [36] | To promote the PD patient’s disease self-management skills, problem-solving capabilities, and knowledge. | Group Sessions: 1. PD overview and medication; 2. Physical functioning; 3. Social Functioning. Held at the clinic site. Taught by interdisciplinary healthcare professional team and 4 trained peer-mentors (people with PD). Included a talk, discussion and individualized mentoring and development of goals & action plans within smaller groups (4-6 participants). 20min break for socializing & relaxation included.  Provided with a ‘toolkit’ including:   - Specific information for the particular session of the intervention. - National Parkinson’s Foundation (NPF) “PD Rainbow Series” booklets (written information about PD, lifestyle and social support resources) - Reference materials related to resources - NPF PD ‘identification card’ - Self-management educational materials | 2-hour weekly sessions over 3 consecutive weeks  *Assessed at the start and end of the programme +4week follow-up* | Standardised programme but with discussion depends on participants. No modifications reported. |
| **Early Management Programme**  Gruber [50] | Intergrates self-management principles with a focus on exercise to improve health status and promote active living | Delivered by a physiotherapist and trained volunteer facilitator who has graduated from an earlier EMP.  Sessions comprise:  1^st^ hour – non-didactic interactive discussion of topics.  2^nd^ hour – learning & practicing specialized exercises for PD.  Discussion topics: introduction, why exercise, relaxation, goal setting, coping with change, Parkinson’s 101, Medical treatment, healthcare professionals, walking, speech & swallow, healthcare consumer, relationships, mind, emotions & behavior, your support team, pain, sleep & relaxation, making daily living easier, review & wrap up.  Exercises: “Axial Mobility Program” – strength, posture & balance. Aerobic exercise discussed and encouraged outside of the programme.  Personal goal setting: short term goals set every 2weeks and 1 long term goal for the programme. | X2/week 2-hour sessions over 8 weeks  *Assessed at the start and end of the programme.* | Standardised programme but with discussion depends on participants. No modifications reported. |
| **Stanford Chronic Disease Self-Management Programme (CDSMP)** Pappa, Nelson [40, 45] | To practise the principles of self-efficacy for managing their chronic disease.  *Studying feasibility* | Companion book provided (Pappa)  Structured programme composed of education and support group elements. Covers problem-solving; decision-making; resource utilization; patient–provider partnerships; and action plans. Sessions interactive encouraging sharing experience. Socialising encouraged in a 20-minute break mid each session.  Nelson - Delivered by 2 psychologists one had PD. *Location of intervention delivery not stated.*  Pappa: 4-17 people per group, location APDA or university. 2 Trained facilitators.  Training for CDSMP is standard, certified & involves a 1--week didactic and experience-based workshop session. | 6 x weekly sessions:  90-120 mins sessions (Nelson)  270 mins sessions (Pappa)  *Assessed at the start and end of the programme + 6months follow-up in Nelson study.* | Discussions would depend on the participants’ specific contributions.  As the workshop advanced and investigators became more familiar with the course and participants, they “recognised the needs for more content flexibility regarding PD information and greater attention to the physical & emotional impact of disease progression”.  No reported modifications. Monitored by attendance records. |
| **Propath (health promotion programme)**  Montgomery [35] | To improve health confidence, provide information and support, improve physical function through exercise, work with physician to optimise medical treatment and compliance. To improve functional outcome using an educational strategy. | Provided free for 9-months in US.  One-page questionnaires mailed to participants at 0, 2, 4 and 6months. From each, a computer generated individualized recommendation letter and progress report were provided to patients with pamphlets and educational materials.  E.g. recommendations for exercise, tailored by age, disease severity, comorbidity, present exercise level, diet, compliance, side effect control and information about dealing with their specific reported problems.  Summary report provided to physician, with suggestions for their consideration (e.g. medication changes).  Reading level was mostly maintained as 8^th^ grade level. | 4 iterations, every 2months, over 6 months  *Assessed at the start and end of the programme.* | Protocolled tailoring of information and recommendations as described. No modifications reported. |
| 1. **Self-management training as above, combined with other therapies** | | | | |
| **Self-management rehabilitation programme** (2 “doses”) Tickle-Degnen [29] | To improve HrQoL  *+ to determine effective “dose”* | 2 intervention conditions based on time.  Intervention is interdisciplinary rehabilitation with self-management:  Group sessions included physical exercises, speech exercises, functional training and a discussion about self-management strategies. Participants assess their problems and set action plans. They were taught problem solving strategies. Team helped them translate these into habits.  27hr condition: also received a ‘transfer of training’ session in their home or community.  18hr condition: also received a student facilitated social group session in the clinic: ice-breaker activities, refreshments, conversation & sharing of hobbies & interests.  Therapists were trained and supervised for consistency. Standardized, manualized intervention.  Physical therapist led all sessions, and occupational therapist + speech & language therapist each participated in half, assisted by therapy students.  Manuals including photographs were provided. | 18h group:.x2 1.5hour group sessions + 1.5hour social.  27hr group: x2 1.5-hour group sessions + 1.5hour home/community session per week.  For 6weeks.  *Follow-up immediately post; at 2months and at 6months.* | The strategies & action plans were tailored to the individual, but the process was manualized.  No reported modifications. Monitored by attendance records.  *Medication adherence monitored via clinical nurse specialist phone calls half-way through the intervention, at completion and at 2- and 6-months follow-up.* |
| **Exercise + self-management programme** Sajatovic [25] | Increase engagement in exercise and improve symptoms of depression  *Studying feasibility & effect of different delivery methods* | 2 delivery methods:   1. Enhanced Exercise Therapy for PD (EXCEED): Manualized group self-management sessions, co-led by a Nurse + trained-peer (person with PD and depression), plus manualized exercise programme led by a certified personal trainer. After the intervention, individuals continued to exercise on their own. 2. Self-guided chronic disease self-management plus exercise (SGE): a single initial in-person group orientation, followed by flexible free access to the fitness facility plus access to the fitness instructor as needed. Asked to exercise the specified amount following written instructions for the exercise programme; weekly phone calls to self-report. Self-management component: provided with written materials, which they access independently, self-paced.   Self-management component: focus on problem solving and goal setting. Exercise: 20-mins fast-paced low-resistance cycling, 20mins progressive sequence resistance band strength training. | 3x /week 1-hour sessions for 12 weeks  *Assessed at the start and end of the programme + follow-up at 24weeks.* | No reported modifications. Monitored by attendance records & self-reported activity during weekly telephone call. |
| **PD Wellbeing Program**  Horne [22]  &  Li [21] | To empower people with PD to make proactive health and lifestyle changes to self-manage their disease.  *+ evaluating long term behaviour change, specifically mood & exercise* | Each session: 1-hour education then 1-hour/1-hour, 10-mins exercise class, then 20/30-mins socialising over a meal, ended with information about continuing to exercise and signposting to local community classes.  Education delivered by multidisciplinary professionals (physiotherapist, exercise physiologist, rehabilitation specialist, dietician, speech pathologist and social worker), exercise class supervised by a physiotherapist and exercise physiologist.  Education topics: PD diagnosis, medication, exercise, nutrition, communication, sleep & fatigue, falls, freezing, posture & stress.  Information booklet provided. Light meal provided.  Exercise class: delivered as group circuit classes. balance, cardiorespiratory fitness, strength, power and large amplitude movements.  Delivered in the day hospital.  Max 6 participants per class. | Twice-weekly sessions for 5 weeks.  *Follow-up at end of programme (6weeks) and 1 year.* | Participants had input into the type of exercises performed in consultation with the treating therapists. Standard exercises were adapted to meet individual needs, preferences and capability.  No modifications reported. |
| **Telephone based, self-management focussed CBT**  Dobkin [31] | To improve mood- and health-related outcomes, through training coping skills to ameliorate depression and emphasising strategies for optimising PD care. | Manual-guided T-CBT: targeted negative thoughts and behaviours, guided by the Chronic Care Model to enhance PD self-management. Primary intervention targets included PD-specific concerns and thoughts blocking PD self-management. Modules: behavioural activation, cognitive restructuring, anxiety management, and sleep hygiene. Manual also acted as participant workbook.  +Care partners received telephone-based coaching in how to support the participant use the CBT skills between sessions.  All conducted remotely.  Given by Masters-level therapists, supervised by a psychologist/Professor of psychiatry. | Weekly for 10 one-hour sessions +/- ongoing 6month follow-up, max 16sessions  *Assessed at the start and end of the programme plus the 6month follow-up.* | Manual based but personalised to individuals’ symptoms and preferences.  No reported modifications. Monitored by participation calls being logged. |
| **ESSENCE mindfulness & self-management programme** Advocat [28] | To improve PD-associated function and wellbeing | Facilitated group sessions composed of:   - Facilitator-led 5-20min mindfulness practice (including specific techniques such as body scan, attention to breath, and letting go of competing or negative thoughts) - Introduction to an element of the ESSENCE framework (education about the condition & lifestyle factors; stress management; spirituality; exercise; nutrition; connectedness; environment) - Open group discussion. - Refreshments provided   3-disc CD pack with guided mindfulness practices and printed information was given (free) to participants for independent use outside of the sessions.  Setting: inner-urban suburbs; venue not specified. | Weekly 2-hour sessions over 6 weeks.  *Assessed at the start and end of the programme + 6month follow-up.* | No reported modifications. Monitored by attendance records and sessions observed by a research assistant. |
| **Integrated Care Network**  Mestre[49] | To integrate care and self-management support, in a community-centred pragmatic care model. It aims to increase accessibility to existing local care resources.  +Feasibility | Multi-component:   - Patient education: evidence based information ‘tip sheets’ - Self-management: Assess, advise, agree, assist & arrange – involves shared decision making, planned care visits and structured follow-up. - Care navigation: network of healthcare resources with review of appropriateness for PD and optimisation of referral pathways.   Delivered by a trained “Clinical Care Integrator” (CCI), corresponding to a specialist PD nurse.  Participants complete online questionnaires ahead of appointment. Then initial visit with CCI: define personal care plan, care priorities and care goals. Telephone review +/- further support; optional follow-up in-person visit, then final closing visit. CCI contactable by telephone or email outside of appointments. | 6months:  Initial visit (90mins); 1month telephone call, optional 3month follow-up visit, closing visit at 6months.  *Assessed at the start, midpoint and end of the programme.* | Standard structure but variation according to the inidivuals’ needs & priortiies. No modifications reported.  The CCI documented the duration of encounters (in-person and phone call visit) and number of other communications. |
| 1. ***Specific Self-Management Skill: Self-Monitoring*** | | | | |
| **Parkinson’s Tracker App (PTA)** Lakshminarayana [26] | Digitally supporting self-management to improve medication adherence primarily but also quality of life, quality of consultations and symptom control. | Given instructions: Download the app within 1day; set up medication reminders and use the app once a day or if not possible at least on alternate day. Telephone call to check if they had downloaded the app & address any difficulties with the app.  PTA is a digital self-management and treatment adherence tool. It includes self-monitoring of 10 measures, a dexterity & cognitive game to self-monitor, a function to generate a report of self-monitored measures, self-set reminders for medication, and information about PD from charities.  Self-monitoring data is used at their clinical appointment.  Delivered from the clinic, with self-monitoring at home. Clinician gave above instructions & telephone call as above. | Telephone call 2weeks after instruction to download.  16 weeks of self-monitoring  *Assessed at the start and end of the programme.* | Delivery is standard but the use of the app is up to the individual.  No reported modifications.  App usage data was recorded (days used and data input) |
| **“UpRight” posture detection device with feedback** Van Wegen [44] | To improve stooped posture  *Studying feasibility* | “UpRight device” provided. It gives vibro-tactile feedback to self-correct stooped posture.  Device is initially applied & calibrated by the assessor. Instructions for applying it, on useage and on correcting posture according to the feedback was provided. This was by 2 trained assessors alongside an illustrated manual. Phone contact with assessors for proper handling.  Conducted in the patient’s home. | 2 weeks of monitoring with the device. Worn throughout the daytime. “regular” phone contact.  *Assessed at the start and end of the programme.* | Delivery is standard but the use of the device is up to the individual.  Useage data collected from the device. |
| **Digital physical activity tracker + online support group**  Hermanns [47] | To improve self-efficacy for physical activity and quality of life.  + feasibility & acceptability | A wrist-worn physical activity tracker (Fitbit Alta HR) was provided, along with a tablet device (iPad Wi-Fi 32Gb, with instructions). The tracker utilizes an accelerometer and gives real-time feedback of activity. It connected to the iPad and can connect to an individual’s computer or smartphone too, to view data. The iPad was also for viewing online exercise videos (including Specific large muscle exercise applications plus instructions for participation and 3 pre-loaded videos) and accessing the online support group (a private forum for sharing information & resources).  Pre-loaded exercise videos: seated warm-up + two for balance, rigidity & gait. Videos showed a physical therapist performing the exercises. | Asked to exercise 3x/week and engage with support group at least 3x/week.  Total 12-weeks.  *Assessed at the start and end of the programme.* | Standard instructions & videos provided, no tailoring described. No reported modifications. Monitored by the tracker and digital data. |
| 1. **Self-management of individual clinical features of PD** | | | | |
| **“What? Me Worry!?!” online self-help guided bibliotherapy** Lawson [27] | To improve worry | Participants provided with a booklet based on the online self-help resource “What? Me Worry!?!” and asked to read one per week and attempt accompanying tasks and exercises. Telephone contact to discuss progress and answer questions.  Modules: overview of general anxiety, overview of worry, negative beliefs (1&2), positive beliefs, challenging worries, letting go of worries, accepting uncertainty, problem solving, relaxation and self-management. | One module per week for 8-weeks.  Telephone calls every 2 weeks.  *+ Follow-up at 3months* | Standardized programme but discussion based on the individual.  No reported modifications. Monitored by telephone calls – participants reported progress. |
| **Saliva cueing device**  McNaney [38] | To improve severity and frequency of drooling.  *+feasibility & acceptability of device use* | Verbal + practical tutorial on how to use the cueing device, in participants own home. Asked to use the device at *different* times to the diary of symptoms being used as an outcome measure.  Device is a wrist-worn digital cueing device that delivers a silent vibratory cue once per minute when switched on.  Information about how frequency of swallowing can improve drooling symptoms given (to control group to). | Advised 1hour/day for total of 4 weeks.  *1week diary assessment at the start, mid-point, end of the programme and 4weeks later.* | No reported modifications. Monitored by participant diaries. |
| **Group communication therapy with self-management strategies** Jordan [46] | To /improve communication skills & compensatory strategies  *+to assess delivery method (supervised volunteers)* | Delivered in nursing homes.  Trained volunteers delivered the programme under supervision of qualified Speech & Language Therapist (SLT).  Variety of presentation styles: oral, audio-visual and practical activities. Format: information giving, discussion and practice of new skills. Supplementary written materials were also provided. Core components: communication process, comprehension, expression and social communication.  Volunteers from advertising and local support groups, received 2x 4hours training sessions about communication disorders and the elderly pus specific disease processes (e.g. PD), plus familiarising with the programme content and the nursing home. Written information and a script provided to volunteers.  SLT observed but did not participate, then debriefed after. | Weekly 2-hour sessions over 6 weeks  *Assessed at the start and end of the programme.* | “Timeframe was flexible to allow for individual variation”  Monitored through observation by the SLT |
| 1. **Self-management of specific treatments, i.e. self-guided treatment programmes** | | | | |
| **Medication /dopamine education** Grosset [24] | To improve medication adherence | Verbal and written (1xA4 sheet) provided on the Continuous Dopaminergic Hypothesis plus tailored written guidance on optimal medicine timing for their drug regimen.  This counselling was provided by one investigator (a GP with special interest in neurology)  Monitoring of medication adherence was by electronic pill bottles.  *Location of intervention delivery not stated.* | Implied single time point for education but not specified.  *Study monitored 3months pre- and 3months post- education.* | Information about optimal medication timing was tailored to the individual by the clinical investigator. No reported modifications. Monitored by number given information. |
| **Self-managed exercise**  Lun [39] | To improve motor symptoms of PD.  *+ compare self-supervised vs physiotherapist supervised programme* | Exercise programme: Physiotherapist-supervised or self-supervised. All given an illustrated instruction booklet.  Self-supervised group had an introductory session with the physiotherapist first then completed the programme independently at home.  Session composition: 20‐minute cardiovascular warm‐up, then 8 × 5 minute exercise stations, ending with 10 minutes of stretching and cool down. Exercisesin 5 positions, working on dual tasking, extension, rotation, reaching, stepping and symmetrical gait.  Physiotherapist was a specialist in PD (same person both groups)  Location of physiotherapist supervised group not stated. | Twice weekly, 60 mins sessions.  Intro session 60 mins.  8-weeks  *Assessed at the start and end of the programme + 8-weeks post was monitored.* | No specified tailoring described.  No reported modifications. Monitored by attendance records for face-to-face sessions and self-reported exercise journals for home group. |
| **Self-managed exercise at home**  Dereli [30] | Improve QoL  *+ compare self-supervised vs physiotherapist supervised programme* | Group exercise plus patient education programme or self-guided exercise plus patient education programme.  Exercises that needed assistance:  > performed in the exercise unit for physiotherapist group  > self models of these for the self-guided group  Patient education programme: performed individually for every participant: information about the diease, rehabilitation, exercises and how to perform them. Accompanying illustrated leaflet with disease and exercise information.  Physio group – done in the exercise unit at the university.  Self-guided group – at home. Motivated by telephone calls from the same physio. | 3x/week for 10 weeks  Sessions were in the mornings, lasting 45 minutes.  Telephone calls to home group were weekly.  *Assessed at the start and end of the programme.* | No tailoring reported. No reported modifications. Monitored by attendance records for the physio group & self-report during telephone calls for the self-guided group. |
| **Home based exercise**  Atterbury [34] | To improve balance.  *To compare home based with therapist supervised balance programme.* | Home based: provided with 8 DVDs with clear instructions and safety guidelines.  Exercise sessions: 10mins warm-up, 15-40mins balance training, 10min cool down with relaxation techniques. DVD instructs caregiver to give verbal kinasthestic and physical tactile cues. Encouraged to use alternative (e.g. somatosensory) strategies to improve balance and gait. Each week focussed on posture, base of support & centre of gravity and progressed to dynamic and functional balance activities incorporating cognitive-motor tasks.  *Comparator: qualified clinical exercise therapist supervised group sessions (4-8 other participants) at a local hall, same frequency.* | X3/week 40-60minute sessions over 8weeks.  *Assessed at the start and end of the programme.* | Protocolled programme. No modifications described. Adherence not discussed. |
| **Home based exercise**  King [37] | To improve mobility.  *To compare home based with individualized therapy and group therapy sessions.* | Sensori-motor Ability Boot Camp (ABC) for all delivery methods: targets basic postural systems. 6 stations: Tai chi, boxing, lunges, kayaking, agility course and pilates.  Home group met the physical therapist once to receive their individualized home exercise program – exercise level based on ability to conduct the exercises safely at home and kept constant. Handouts provided. Therapist were highly experienced in PD and ABC. Therapists rotated between cycles to avoid bias.  *Individual therapy: at the outpatient rehabilitation centre, tailored progression.*  *Group therapy: at the wellness center at the University, tailored progression.*  Monitored by recording rate of perceived exertion after each session. | X3/week 60-minute sessions for 4weeks.  *Assessed at the start and end of the programme.* | Protocolled programme. No modifications described. Adherence in home group not explicitly discussed. |
| **Self-managed exercise programme** Collett [18] | To provide support and overcome barriers whilst encouraging self-management incorporating evidence for safe effective exercise | Booklet led exercise sessions: 30 mins of aerobic training and  30 mins resistance.  Support was an initial face-to-face introductory session and monthly progress sessions. Participants could contact for information and advice in between.  The booklet included a diary for monitoring by the participant.  Travel & gym costs covered by the study.  In community public leisure facilities: participant could choose from participating locations.  Support by a specialist exercise practitioner or a physiotherapist. They received training on how to deliver the prescribed exercise. Leisure facility staff were fully informed about the study and that the participants were following a prescribed exercise program. | 48 sessions over a 24 week period (2x a week). Each 60minutes. *Assessed at the start, mid-point and end of the programme + follow-up at 12months.* | Personalised according to specific protocols: tailoring aerobic intensity to maximal heart rate and resistance based on starting ability. No reported modifications. Monitored by participants recording in their booklets. |
| **Self-managed handwriting exercises** Collett [17] | As the control for above, after consultation people with Parkinson’s, who identified that handwriting was an important issue and would have the desired effect engaging people through the intervention period | Booklet led handwriting exercise sessions: printed workbook which also included monitoring. Exercises included warm-up, writing activities and exercises (e.g. rolling putty)  Support was an initial face-to-face introductory session and monthly progress sessions. Participants could contact for information and advice in between. The same staff as above**.**  Exercises also used ‘play dough’ putty, clothes pegs, lolly sticks and a jar, and a soft tennis ball. Materials were provided. | 48 sessions over a 24 week period (2x a week). Each 60minutes.  *Assessed at the start, mid-point and end of the programme + follow-up at 12months.* | No formal tailoring all followed the same workbooks. Feedback given at the monthly review.  No reported modifications. Monitored by participants recording in their booklets. |
| **“Park-in-Shape”**  Van der Kolk [33] | To relieve motor symptoms of PD | Home based aerobic exercise: cycling on a stationary home trainer, enhanced with virtual reality software and real-life videos (providing a so-called “exergaming” experience) plus a motivational app and remote supervision.  Instructed to do 30–45 min (30 min aerobic + 15 min warming up and cooling down) at least three times per week, at a target heart rate zone, which was gradually increased for goal setting as patients became fitter. Direct feedback of Heart Rate via computer. App included training instructions, tips for optimal training effect, support from loved ones via messages, and the opportunity to monitor their progress. Coaching & supervision: One manualised home visit then remote supervision by fortnightly telephone call.  Control: home based stretching; also had app and remote supervision. Instructed to do three times per week for 30 min per session. | 30–45 min training three times per week for 6 months  *Assessed at the start and end of the programme.* | Manualised coaching, fixed instructions and protocolled escalation of exercise targets, but actual participation would depend on the individual. No reported modifications. Monitored by digital record of exercise bike use. |
| **Conduction Exercise & Self-acupressure**  Yuen [16] | Improvement of motor and non-motor symptoms. | Conduction Exercise (CE) plus Self-Acupressure (SA). CE is a set of manoeuvres combining rhythmic breathing and bodily movements. SA, is the massage of acupoints.  Exercise programme taught in weekly sessions plus instructed to practise every day at least once and twice at maximum at home.  Taught by Research Assistant. Location of teaching not clear.  Aids: An illustrated booklet containing all 14 steps of CE and SA.  Control: 2 sessions of “health related talk” | 8 weekly sessions 60-90 mins each.  *Assessed at the start, midpoint and end of the programme.* | Feedback obtained from participants & caregivers after commencement of the study to make it “more patient-friendly” – not clear when changes were made.  Monitored by participants keeping a record of their home practice.  Extra time was given to those who failed to meet the standard. |
| **Home based balance training**  Esculier [51] | To improve balance and functional ability. | 2 members of the research team installed the equipment, explained the programme and supervised the first session.  Equipment: Wii console, game and balance board.  Also given a logbook to record game scores, satisfaction and give additional information about the programme & safety.  Sessions prescribed: 30mins with a Wii Fit game with the balance board (4 exercise sections, 1 yoga, and 1 aerobic) and 10mins with the Wii Fit sports game (golf or bowling).  Training progression guided by the game: good performance lead to increased difficulty.  Motivational telephone calls by research team each week and encouraged to contact the team with questions or technical problems. | 40mins sessions, 3days/week for 6 weeks (18 in total).  Weekly phone call.  *Assessed at the start, midpoint and end of the programme.* | No changes made in the games to maintain uniformity. Tailoring according to performance as described. Monitoring by logbook. No modifications reported. |

PD = Parkinson’s Disease
