# Supplementary material for: Systematic review and meta-analysis of clinical effectiveness of self-management interventions in Parkinson’s disease
Source: BMC Geriatr. 2022 Jan 11;22:45. doi: 10.1186/s12877-021-02656-2 (PMC8753859; doi:10.1186/s12877-021-02656-2)
Supplement: Supplementary file 3 — Additional file 3. Risk of Bias Assessment. [file 12877_2021_2656_MOESM3_ESM.docx]

**ADDITIONAL FILE 3: Risk of Bias Assessment**

**ROB2 Tool for RCTs**

| **Domains** | **Signalling questions** | Dobkin | Navarta Sanchez | Yuen | Van der Kolk | Atterbury | Collett (x2) | Lakshminarayana | Sajatovic | Advocat | King | Lawson | A’Campo | Dereli | Tickle-Degnen | Grosset | Pearl-Kraus | Montgomery | McNaney |
| --- | --- | --- | --- | --- | --- | --- | --- | --- | --- | --- | --- | --- | --- | --- | --- | --- | --- | --- | --- |
| 1.Randomization process: | 1.1 Was the allocation sequence random? | Y | PN | Y | Y | PN | Y | Y | PY | Y | Y | Y | PY | PY | Y | Y | Y | PY | PN |
|  | 1.2 Was the allocation sequence concealed until participants were enrolled and assigned to interventions? | Y | N | Y | Y | NI | Y | Y | PY | PY | Y | Y | Y | N | Y | Y | Y | PY | PN |
|  | 1.3 Did baseline differences between intervention groups suggest a problem with the randomization process? | N | PY | N | N | PY | PN | N | PY | N | N | N | PN | N | PN | N | PN | PN | N |
|  | Overall risk of bias | Low | High | Low | Low | High | Low | Low | Some concern | Low | Low | Low | Low | High | Low | Low | Low | Low | High |
| 2. Deviation from Intended intervention:  (Intention-to treat-) | 2.1. Were participants aware of their assigned intervention during the trial? | Y | Y | Y | PN | PY | Y | Y | Y | PN | Y | PY | Y | Y | Y | N | PN | NI | PN |
|  | 2.2. Were carers and people delivering the interventions aware of participants' assigned intervention during the trial? | Y | Y | PN | PY | PY | Y | Y | PY | PN | Y | PY | Y | Y | Y | PN | PY | NI | PY |
|  | 2.3. If Y/PY/NI to 2.1 or 2.2: Were there deviations from the intended intervention that arose because of the trial context? | N | N | PN | N | PN | PN | PN | N | PN | PN | N | N | N | N | NA | N | NA | Y |
|  | 2.4 If Y/PY to 2.3: Were these deviations likely to have affected the outcome? | NA | NA | NA | NA | NA | PN | NA | NA | NA | NA | NA | NA | NA | NA | NA | NA | NA | PN |
|  | 2.5. If Y/PY/NI to 2.4: Were these deviations from intended intervention balanced between groups? | NA | NA | NA | NA | NA | NA | NA | NA | NA | NA | NA | NA | NA | NA | NA | NA | NA | PN |
|  | 2.6 Was an appropriate analysis used to estimate the effect of assignment to intervention? | Y | Y | PY | Y | Y | PY | Y | Y | Y | PY | Y | Y | PY | Y | Y | PY | PY | PN |
|  | 2.7 If N/PN/NI to 2.6: Was there potential for a substantial impact (on the result) of the failure to analyse participants in the group to which they were randomized? | NA | N | NA | NA | NA | NA | N | NA | NA | NA | NA | NA | NA | NA | NA | NA | NA | PN |
|  | Risk-of-bias judgement | Low | Low | Low | Low | Low | Low | Low | Low | Low | Low | Low | Low | Low | Low | Low | Low | Some concern | High |
| 3.Missing outcome data | 3.1 Were data for this outcome available for all, or nearly all, participants randomized? | Y | PY | N | Y | PN | Y | N | Y | PN | Y | PN | Y | Y | Y | PN | Y | NI | Y |
|  | 3.2 If N/PN/NI to 3.1: Is there evidence that the result was not biased by missing outcome data? | NA | NA | PN | NA | PN | NA | PN | NA | PN | NA | PY | NA | NA | NA | PN | NA | N | NA |
|  | 3.3 If N/PN to 3.2: Could missingness in the outcome depend on its true value? | NA | NA | PY | NA | Y | NA | Y | NA | PY | NA | NA | NA | NA | NA | PY | NA | NI | NA |
|  | 3.4 If Y/PY/NI to 3.3: Is it likely that missingness in the outcome depended on its true value? | NA | NA | PN | NA | PY | NA | PY | NA | PY | NA | NA | NA | NA | NA | PY | NA | NI | NA |
|  | Risk-of-bias judgement | Low | Low | Some concern | Low | High | Low | High | Low | High | Low | Some concern | Low | Low | Low | High | Low | High | Low |
| 4.Measurement of Outcome: | 4.1 Was the method of measuring the outcome inappropriate? | N | PN | N | N | PN | N | PN | PN | PN | PN | N | N | N | N | PN | PN | PN | PN |
|  | 4.2 Could measurement or ascertainment of the outcome have differed between intervention groups? | N | PN | N | N | PN | N | PN | N | PY | PN | N | N | N | N | PN | PN | PN | PN |
|  | 4.3 If N/PN/NI to 4.1 and 4.2: Were outcome assessors aware of the intervention received by study participants? | PY | Y | PY | N | PY | PY | Y | PY | PY | PY | PY | PY | PY | PY | PN | PN | PY | PY |
|  | 4.4 If Y/PY/NI to 4.3: Could assessment of the outcome have been influenced by knowledge of intervention received? | PY | PY | PY | NA | PY | PN | Y | PY | PY | PY | PY | PY | PY | PY | NA | NA | PY | PY |
|  | 4.5 If Y/PY/NI to 4.4: Is it likely that assessment of the outcome was influenced by knowledge of intervention received? | PN | PN | PN | NA | PN | PN | PY | PN | PN | PN | PN | PN | PN | PN | NA | NA | PN | PN |
|  | Risk-of-bias judgement | Some concern | Some concern | Some concern | Low | Some concern | Some concern | High | Some concern | Some concern | Some concern | Some concern | Some concern | Some concern | Some concern | Low | Low | Some concern | Some concern |
| 5. Selection of reported result: | 5.1 Were the data that produced this result analysed in accordance with a pre-specified analysis plan that was finalized before unblinded outcome data were available for analysis?  Is the numerical result being assessed likely to have been selected, on the basis of the results, from. | PY | PY | PY | PY | PN | PY | PY | N | PY | PY | PY | PY | PY | PY | PY | PY | PY | NI |
|  | 5.2. ... multiple eligible outcome measurements (e.g. scales, definitions, time points) within the outcome domain? | PN | PN | PY | PN | PN | PN | PN | PY | PN | PN | PN | PN | PN | PN | PN | PN | PN | PN |
|  | 5.3 ... multiple eligible analyses of the data? | PN | PN | PN | PN | PN | PN | PN | PY | PN | PN | PN | PN | PN | PN | PN | PN | PN | PN |
|  | Risk-of-bias judgement | Low | Low | Low | Low | Low | Low | Low | High | Low | Low | Low | Low | Low | Low | Low | Low | Low | Some concern |
| Overall risk of bias: |  | Some concern | High | Some concern | Low | High | Some concern | High | High | High | Some concern | Some concern | Some concern | High | Some concern | High | Low | High | High |

**ROBIN-I Tool for Non-randomized Studies of Interventions:**

| **Domains** | **Signalling questions** | Hellqvist | Lyons | Pappa | Lun | Lindskov | Li | Mestre | Horne | Van Wegen | Hermanns | Esculier | Nelson | Gruber | Macht | Simons | Sunvisson | Jordan |
| --- | --- | --- | --- | --- | --- | --- | --- | --- | --- | --- | --- | --- | --- | --- | --- | --- | --- | --- |
| **Bias due to confounding** | 1.1 Is there potential for confounding of the effect of intervention in this study?  **If N/PN to 1.1:** the study can be considered to be at low risk of bias due to confounding and no further signalling questions need be considered | PY | PY | PY | Y | PN | PY | PY | PY | PY | PY | PN | PY | PY | PY | PY | PY | PY |
|  | **If Y/PY to 1.1**: determine whether there is a need to assess time-varying confounding: |  |  |  |  |  |  |  |  |  |  |  |  |  |  |  |  |  |
|  | 1.2. Was the analysis based on splitting participants’ follow up time according to intervention received?  **If N/PN**, answer questions relating to baseline confounding (1.4 to 1.6)  **If Y/PY**, go to question 1.3. | N | N | N | PN | NA | N | N | N | N | N | NA | N | N | N | N | N | N |
|  | 1.3. Were intervention discontinuations or switches likely to be related to factors that are prognostic for the outcome?  **If N/PN**, answer questions relating to baseline confounding (1.4 to 1.6)  **If Y/PY**, answer questions relating to both baseline and time-varying confounding (1.7 and 1.8) | PY | NA | NA | PN | NA | NA | NA | NA | NA | NA | NA | NA | NA | NA | NA | NA | NA |
|  | **Questions relating to baseline confounding only** |  |  |  |  |  |  |  |  |  |  |  |  |  |  |  |  |  |
|  | 1.4. Did the authors use an appropriate analysis method that controlled for all the important confounding domains? | PN | PN | PN | PN | NA | Y | PN | Y | PN | PN | NA | PN | PN | PN | PN | PN | PN |
|  | 1.5. **If Y/PY to 1.4**: Were confounding domains that were controlled for measured validly and reliably by the variables available in this study? |  |  |  | NA | NA | Y | PY | Y | PN | NA | NA | PY | PN | PY | PY | PY | PN |
|  | 1.6. Did the authors control for any post-intervention variables that could have been affected by the intervention? | PN | NA | N | NA | NA | N | N | N | N | N | NA | N | N | N | N | N | N |
|  | **Questions relating to baseline and time-varying confounding** |  |  |  |  |  |  |  |  |  |  |  |  |  |  |  |  |  |
|  | 1.7. Did the authors use an appropriate analysis method that controlled for all the important confounding domains and for time-varying confounding? | PN | NA | NA | NA | NA | NA | NA | NA | NA | NA | NA | NA | NA | NA | NA | NA | NA |
|  | 1.8. **If Y/PY to 1.7**: Were confounding domains that were controlled for measured validly and reliably by the variables available in this study? |  | NA | NA | NA | NA | NA | NA | NA | NA | NA | NA | NA | NA | NA | NA | NA | NA |
|  | **Risk of bias judgement** | Mod | Mod | Mod | Serious | Low | Low | Mod | Low | Mod | Mod | Low | Mod | Mod | Mod | Mod | Mod | Mod |
| **Bias in selection of participants into the study** | 2.1. Was selection of participants into the study (or into the analysis) based on participant characteristics observed after the start of intervention?  **If N/PN to 2.1:** go to 2.4 | N | N | N | PN | N | PN | N | PN | N | N | N | N | N | N | N | PY | N |
|  | 2.2. **If Y/PY to 2.1**: Were the post-intervention variables that influenced selection likely to be associated with intervention? | NA | NA | NA | NA | NA | NA | NA | NA | NA | NA | NA | NA | NA | NA | NA | N | NA |
|  | 2.3 **If Y/PY to 2.2**: Were the post-intervention variables that influenced selection likely to be influenced by the outcome or a cause of the outcome? | NA | NA | NA | NA | NA | NA | NA | NA | NA | NA | NA | NA | NA | NA | NA | NA | NA |
|  | 2.4. Do start of follow-up and start of intervention coincide for most participants? | Y | Y | Y | PY | PY | Y | Y | Y | Y | Y | PY | Y | Y | Y | Y | Y | Y |
|  | 2.5. **If Y/PY to 2.2 and 2.3, or N/PN to 2.4**: Were adjustment techniques used that are likely to correct for the presence of selection biases? | NA | NA | NA | NA | NA | NA | NA | NA | NA | NA | NA | NA | NA | NA | NA | NA | NA |
|  | **Risk of bias judgement** | Low | Low | Low | Low | Low | Low | Low | Low | Low | Low | Low | Low | Low | Low | Low | Low | Low |
| **Bias in classification of interventions** | 3.1 Were intervention groups clearly defined? | Y | Y | Y | Y | Y | Y | Y | Y | Y | Y | Y | Y | Y | Y | Y | Y | Y |
|  | 3.2 Was the information used to define intervention groups recorded at the start of the intervention? | Y | Y | Y | Y | Y | Y | Y | Y | Y | Y | Y | Y | Y | Y | Y | Y | Y |
|  | 3.3 Could classification of intervention status have been affected by knowledge of the outcome or risk of the outcome? | N | N | N | N | N | N | N | N | N | N | N | N | N | N | N | N | N |
|  | **Risk of bias judgement** | Low | Low | Low | Low | Low | Low | Low | Low | Low | Low | Low | Low | Low | Low | Low | Low | Low |
| **Bias due to deviations from intended interventions** | 4.1. Were there deviations from the intended intervention beyond what would be expected in usual practice? | N | N | N | N | N | N | N | N | N | PY | PN | N | PN | PN | PY | N | N |
|  | 4.2. **If Y/PY to 4.1**: Were these deviations from intended intervention unbalanced between groups *and* likely to have affected the outcome? | NA | NA | NA | NA | NA | NA | NA | NA | NA | NA | NA | NA | NA | NA | NA | NA | NA |
|  | **Risk of bias judgement** | Low | Low | Low | Low | Low | Low | Low | Low | Low | Mod | Low | Low | Low | Low | Mod | Low | Low |
| **Bias due to missing data** | 5.1 Were outcome data available for all, or nearly all, participants? | Y | PN | Y | Y | Y | Y | Y | Y | Y | Y | Y | PN | PN | PY | PN | PY | Y |
|  | 5.2 Were participants excluded due to missing data on intervention status? | N | N | N | N | N | N | N | N | N | N | N | N | N | N | N | N | N |
|  | 5.3 Were participants excluded due to missing data on other variables needed for the analysis? | Y | PY | PN | PY | PY | PY | PY | PY | N | N | PY | Y | PY | PY | PY | PN | N |
|  | 5.4 **If PN/N to 5.1, or Y/PY to 5.2 or 5.3**: Are the proportion of participants and reasons for missing data similar across interventions? | Y | N | NA | PY | Y | NA | NA | NA | NA | NA | NA | NA | NA | NA | NA | NA | NA |
|  | 5.5 **If PN/N to 5.1, or Y/PY to 5.2 or 5.3**: Is there evidence that results were robust to the presence of missing data? | PY | PN | NA | PY | NA | PN | PY | PN | NA | NA | PY | PN | PN | NA | N | NA | NA |
|  | **Risk of bias judgement** | Mod | Mod | Low | Low | Low | Mod | Low | Mod | Low | Low | Low | Mod | Mod | Mod | Mod | Low | Low |
| **Bias in measurement of outcomes** | 6.1 Could the outcome measure have been influenced by knowledge of the intervention received? | PY | PY | PY | PY | PY | Y | Y | Y | PY | Y | PN | Y | Y | PY | PY | PY | PY |
|  | 6.2 Were outcome assessors aware of the intervention received by study participants? | Y | PY | Y | Y | PY | Y | Y | Y | Y | Y | Y | Y | Y | Y | PY | PY | Y |
|  | 6.3 Were the methods of outcome assessment comparable across intervention groups? | Y | Y | Y | Y | Y | NA | NA | NA | NA | NA | Y | NA | NA | NA | NA | NA | NA |
|  | 6.4 Were any systematic errors in measurement of the outcome related to intervention received? | N | N | N | PN | N | PN | N | PN | N | N | N | N | N | N | N | N | N |
|  | **Risk of bias judgement** | Mod | Mod | Mod | Mod | Mod | Mod | Mod | Mod | Mod | Mod | Low | Mod | Mod | Mod | Mod | Mod | Mod |
| **Bias in selection of the reported result** | Is the reported effect estimate likely to be selected, on the basis of the results, from... |  |  |  |  |  |  |  |  |  |  |  |  |  |  |  |  |  |
|  | 7.1. ... multiple outcome *measurements* within the outcome domain? | PN | PN | PN | PN | PN | PN | N | PN | N | N | PN | N | N | N | N | N | N |
|  | 7.2 ... multiple *analyses* of the intervention-outcome relationship? | PN | PN | PN | PN | PN | PN | N | PN | N | N | PN | N | N | N | N | N | N |
|  | 7.3 ... different *subgroups*? | PN | PN | PN | N | PN | PN | N | PN | N | N | PN | N | N | N | N | N | N |
|  | **Risk of bias judgement** | Low | Low | Low | Low | Low | Low | Low | Low | Low | Low | Low | Low | Low | Low | Low | Low | Low |
| **Overall bias** |  | Mod | Mod | Mod | Serious | Mod | Mod | Mod | Mod | Mod | Mod | Low | Mod | Mod | Mod | Mod | Mod | Mod |
