# Supplementary material for: Systematic review and meta-analysis of clinical effectiveness of self-management interventions in Parkinson’s disease
Source: BMC Geriatr. 2022 Jan 11;22:45. doi: 10.1186/s12877-021-02656-2 (PMC8753859; doi:10.1186/s12877-021-02656-2)
Supplement: Supplementary file 4 — Additional file 4. Results of Intervention Evaluations. [file 12877_2021_2656_MOESM4_ESM.docx]

**ADDITIONAL FILE 4. Results of Intervention Evaluations**

15 studies included participant evaluation of the intervention through custom questionnaires, the Health Education Impact Questionnaire (heiQ) or qualitative interviews.

Participant feedback regarding the interventions was generally positive. Global ratings of usefulness or benefits from the interventions were reported favourably by the majority of participants for all 11 studies that report on this– these include group-based self-management education and training interventions (including combined with rehabilitation) [20, 23, 25, 28, 36, 41, 55], the two self-monitoring and one cueing digital devices[38, 44, 47], and the integrated care network[48]. 83-97% of participants would recommend the intervention to others for the 3 studies that reported asking this[20, 45, 48]. Usability and acceptability feedback, covered in the 3 studies of devices and one of self-help reading material was positive for the most part but with mixed reviews for the physical activity tracker[47]. Appropriateness or satisfaction with content was reported positively for 2 of the 3 group based self-management education and training interventions reporting on this, with the other finding that less than 50% of participants were satisfied with the topics[45]. The HeiQ tool used to evaluate the National Parkinson’s School, a group based self-management education and training intervention, showed statistically significant improvements post interventions for 2 domains: constructive attitudes and approaches (p=0.003) and skill and technique acquisition (p<0.001).

The studies present limited critique by participants. 3 self-management education and training interventions reported areas for improvement: all reflected the participants’ desire for more time or more topics to be covered[20, 36, 45]. One also identified that the exercises that were introduced to help patients learn and develop new skills were experienced to be difficult by about one-third of the patients[20]. In another study of self-management training combined with rehabilitation some participants found the fixed-time groups challenging, along with transportation and logistical difficulties[25]. The telephone-supported self-guided CBT intervention noted that several participants commented that they would have preferred contact in person rather than by telephone to discuss issues[27]. Study of the physical activity tracker found participants were challenged by the technology. The authors propose multiple one-on-one sessions with each participant and repeated demonstration would be beneficial to assist with navigation in online settings[47].
