# Supplementary material for: Systematic review and meta-analysis of clinical effectiveness of self-management interventions in Parkinson’s disease
Source: BMC Geriatr. 2022 Jan 11;22:45. doi: 10.1186/s12877-021-02656-2 (PMC8753859; doi:10.1186/s12877-021-02656-2)
Supplement: Supplementary file 5 — Additional file 5. GRADE Approach: Evidence Profile for Meta-analysis. [file 12877_2021_2656_MOESM5_ESM.docx]

**GRADE Approach: Evidence Profile for Meta-analysis**

**Question**: What is the effect of Self-Management Training Interventions compared to Control for improving QoL in Parkinson’s?

| **Certainty assessment** | | | | | | | **№ of patients** | | **Effect** | | **Certainty** | **Importance** |
| --- | --- | --- | --- | --- | --- | --- | --- | --- | --- | --- | --- | --- |
| **№ of studies** | **Study design** | **Risk of bias** | **Inconsistency** | **Indirectness** | **Imprecision** | **Other considerations** | **Group based Self-Management Interventions** | **Control without self-management (usual care or information only)** | **Relative (95% CI)** | **Absolute (95% CI)** |  |  |
|  | | | | | | | | | | | | |
| 4 | Randomised Controlled Trials | serious **^a^** | serious **^b^** | not serious ^c^ | serious ^d^ | none ^e^ | 229 | 249 | - | Hedges's g:  -0.17 (-0.56, 0.21) | ⨁◯◯◯ Very low | Important |

^a^Risk of Bias assessed as "High" for Navarta-Sanchez and Montgomery studies; "some concerns" for A'Campo; and “low” for Pearl-Kraus (see Additional File 3) for details

^b^Large variation in effects seen, though confidence intervals are mostly overlapping between studies (but for Navarta-Sanchez and A’Campo). This may be related to the different control arm (information group) in the Navarta-Sanchez study. I^2^=68.49% suggesting substantial heterogeneity.

^C^The selected outcome is applicable as a patient important outcome, and the PICO is generalizable

^d^Most studies have large confidence intervals and number of participants are relatively small

^e^Insufficient number of studies for funnel plot; publication bias undetected
